# Pain Cues in People With Dementia: Scoping Review

**DOI:** 10.2196/75671

**Published:** 2025-11-27

**Authors:** Urška Smrke, Ana Milošič, Izidor Mlakar, Matic Kadiš, Satja Mulej Bratec

**Affiliations:** 1 Faculty of Electrical Engineering and Computer Science, University of Maribor, Koroška cesta 46, Maribor, 2000, Slovenia, +386 2 220 72 67; 2 Department of Psychology, Faculty of Arts, University of Maribor, Maribor, Slovenia

**Keywords:** artificial intelligence, digital monitoring, pain detection, pain, dementia, symptoms, review, screening

## Abstract

**Background:**

Individuals with dementia, especially those in later stages, have difficulties with verbally reporting their experience of pain. This results in both underassessment and undertreatment of pain, signaling the need for better pain recognition in persons with dementia. A promising form of pain assessment is digital monitoring, which can concurrently and more objectively detect and use numerous relevant pain cues.

**Objective:**

This review aimed to identify observable cues of pain, which could be used for digital pain monitoring. A total of 2 research questions (RQs) were formed as we set out to examine which digital cues offered a valid insight into pain in people with dementia (RQ1) and identify how these cues were originally measured (RQ2).

**Methods:**

A standard methodological approach for scoping reviews was used. Relevant research papers were chosen based on SCOPUS and Web of Science databases, and relevant data on pain cues were extracted from all papers that satisfied the inclusion criteria. The gathered data were analyzed using a thematic analysis, which involved categorizing the observable cues into higher-order categories.

**Results:**

Of the 3705 publications identified in the search, 34 satisfied the inclusion criteria and were closely examined. Addressing RQ1, we identified 7 categories of behavioral and physiological cues associated with pain, most frequently facial expressions (20/34, 59%) and body movements or expressions (15/34, 44%). Several subcategories for each main category of pain cues were also identified, each involving between 1 and 28 relevant specific pain cues. Addressing RQ2, 29/34 (85%) studies assessed pain cues via human observation only, while 5/34 (15%) combined human observation with either facial recognition software, PainChek app, or computer vision.

**Conclusions:**

The review provides a comprehensive list of the most relevant cues that signify pain in persons with dementia and offers a foundation for the use of artificial intelligence and digital monitoring for the screening of pain in dementia.

## Introduction

Dementia presents a global health concern, with some estimates suggesting that it affects over 50 million people worldwide, and this figure is predicted to triple by 2050 [1,2]. The course of dementia can span from 5 to 12 years, during which the patient’s ability to function independently gradually decreases with the progression of the disease [3]. With this progression, there is also an increase in communication problems [4]. In a clinical setting, this can lead to the underassessment of many co-occurring health conditions or states, especially for patients with moderate to severe dementia [5,6]. One of these underrecognized states is pain [7]. Pain is frequently experienced by patients with moderate to severe dementia but is often undetected and consequently untreated because of their inability to self-report [8]. Since reliable recognition and assessment of pain are essential for effective treatment, observational pain tools that emphasize observable cues have become increasingly important for the accurate assessment of pain in people with dementia [9].

While self-reporting is generally considered the most reliable method for assessing pain [9], a different approach is needed when assessing patients with dementia. There are numerous observational scales available, such as the Abbey Pain Scale (APS) [10], Doloplus-2 [11], the Pain Assessment Checklist for Seniors with Severe Dementia (PACSLAC) [12], and The Pain Assessment in Advanced Dementia Scale (PAINAD) [13]. The problem with this type of assessment, however, is that the scales have poor or unproven reliability, insufficient evidence for validity, and untested sensitivity to change. Additionally, their implementation in practice is often poor [14,15]. It is usually nurses or caregivers who assess and report on the patient’s pain, and they do not always rely on observational scales. Even when they do, their ratings of pain are not always related to any specific pain behaviors, which results in inadequate pain assessment [16-18].

As an alternative, digital monitoring of pain could be very effective in assessing pain in patients with dementia, as it promises to provide objective evidence of the presence and intensity of pain [5,8,19]. The method could use a combination of different technologies, such as automated facial recognition and analysis, smart computing, affective computing, and cloud computing (ie, Internet of Things) for identifying the presence of pain in patients with advanced dementia [20]. As an example, Atee and colleagues [5] recently developed an electronic Pain Assessment Tool (ePAT), an application that uses facial recognition technology to detect facial microexpressions indicating pain and to record pain-related behaviors. A similar system is also used in health care—Internet of Things—enabled surveillance cameras capture real-time video data and can enhance patient care with features like sentiment analysis and emotion detection [21]. Digital monitoring has the potential to change pain assessment in individuals who are unable to verbalize their inner states, as it can be used by clinicians and caregivers in everyday clinical practice [5,8,20].

A critical foundation for effective digital monitoring is a thorough overview and categorization of cues associated with pain that can be measured using digital technologies (ie, digital cues). These are essential for identifying pain in individuals unable to directly report it themselves. However, despite previous efforts in identifying and categorizing pain cues (eg, by the American Geriatrics Society [22]), which identified broad categories of facial expressions, verbalizations and vocalizations, body movements, changes in interpersonal interactions, changes in activity patterns or routines, and mental status changes, the categorizations of pain cues remain inconsistent and segmented. If we want to develop better, technology-supported ways of monitoring pain, a systematic and thorough set of digital pain cues is needed.

Therefore, the main goal of this paper was to identify specific cues that could help identify pain in patients with dementia by way of digital monitoring. Despite many studies that identify common pain behaviors (eg, [22,23]), there is a lack of specific information about how certain cues are measured, or how identified behaviors are linked with pain. This scoping review aimed to address this gap by identifying pain cues that could potentially be intercepted using artificial intelligence (AI). Among other potential applications, results will be directly used to develop an AI-based pain identification system within the project Artificial intelligence–based health, optimism, purpose, and endurance in palliative care for dementia (AI4HOPE [24]). A total of 2 research questions (RQs) were formed for the purpose of this study. The first aimed to investigate which digital cues offer a valid insight into pain in patients with dementia, and the second focused on determining how these cues were measured. To address the RQs, we conducted a scoping review.

## Methods

### Overview

This scoping review was carried out following the framework established by Arksey and O’Malley [25], which involves 5 key stages: formulating the RQs, finding relevant studies, selecting studies, organizing the data, and finally, synthesizing, summarizing, and reporting the results. The findings from our review are presented in accordance with the PRISMA-ScR (Preferred Reporting Items for Systematic Reviews and Meta-Analyses extension for Scoping Reviews) guidelines for scoping reviews [26,27]. The protocol for the review was established in detail beforehand and no changes were made to it once the study was initiated.

### Identifying the Research Questions

To begin working toward a comprehensive list of digital cues that can help identify pain in people with dementia, 2 RQs were formed. The first RQ, “Which digital cues offer a valid insight into pain in people with dementia,” aimed to identify crucial digital cues that can provide reliable insights into pain experienced by people with dementia. The second RQ, “How are these cues measured?” served to identify tools, methodologies, and techniques used to detect and measure the cues.

### Identifying Relevant Studies

To address the RQs, we used 2 databases, Web of Science and Scopus. These databases were selected due to their comprehensive coverage of peer-reviewed journals, conference proceedings, and other scholarly publications across various disciplines. Only the 2 databases were chosen, considering that this was a scoping review and because this ensured a thorough and reliable search, as outlined by López-Illescas et al [28] and Zhu and Liu [29]. They complement each other well [30] and overlap largely with other databases [31]. We first conducted preliminary test searches to optimize our search string [32]. The main search was conducted on April 11, 2024.

The finalized search string included keywords identifying various types of dementia and cognitive impairment (dementia OR Alzheimer* OR Frontotemporal OR Korsakoff OR Creutzfeldt OR “Posterior Cortical Atrophy” OR “Normal Pressure Hydrocephalus” OR “Chronic traumatic encephalopathy” OR “Lewy Bod*” OR “HIV* neurocognitive disorder” OR “Corticobasal syndrome” OR “neurocognitive disorder”), cues (sign OR signs OR signal* OR cue* OR symbol* OR pattern* OR clue* OR manifestation* OR express* OR feature* OR indicator* OR cue* OR “digital biocue*” OR “electronic biocue*” OR sensor* OR “observable cue*” OR “physiological cue*” OR “behavioral cue*” OR “behavioral data” OR “physiological data” OR index OR indices OR property OR reaction* OR characteristic* OR pattern* OR “behavioral cue*” OR “behavioral data” OR behavior* OR behavior* OR change* OR expression* OR respons* OR observ*), modality of cues (text OR video OR image OR audio OR speech OR language OR paralinguistic OR prosodic OR semantic OR acoustic OR lexical OR facial OR visual OR appearance-based OR vocal* OR written OR verbal OR nonverbal OR conversational OR behavior* OR behavior* OR movement OR soft OR motor OR psychomotor OR somatic OR word* OR social OR mood OR eat* OR sleep* OR physiologic* OR body OR communicat* OR noise* OR breath* OR activit*) additional specific observable cues (“sore areas” OR rubbing OR bracing OR restlessness OR consolability OR appearance OR “startle response” OR trembling OR palpitation* OR hyperventilat* OR sweat* OR dizziness), and excluding studies with nonhuman subjects (mouse OR mice OR rat* OR animal*).

Inclusion criteria for the initial search were for the paper to be (1) in English and (2) a peer-reviewed empirical study, published in scientific journals or conference proceedings as full papers (excluding abstracts-only proceedings, preprints, and unpublished work). We restricted the search to publications in the English language, considering the language proficiency of the authors as well as the evidence that this does not have a significant impact on the conclusions of reviews [33]. We did not restrict the papers based on the year of publication.

### Study Selection

All identified citations were exported to Microsoft Excel spreadsheets. The study selection procedure was then performed in 2 review phases. In the first phase, 6 researchers (IM, US, SMB, AM, MK, and another colleague) independently screened titles and abstracts to determine eligibility. A small sample of papers was screened before the first phase and reviewed by 2 researchers (SMB and US) to ensure the agreement on inclusion criteria among all the researchers. In the second phase, the same authors reviewed full articles, each reviewing a different set of articles from phase 1. In the second phase, we excluded records based on predetermined exclusion criteria, that is, those that (1) were not an empirical study (eg, reviews and meta-analyses), (2) were not performed on human participants, (3) were not focusing on adults, (4) did not report associations between observable cues and pain, (5) focused on self-reported cues, (6) focused on cues that required specialized equipment (eg, magnetic resonance imaging [MRI] and electroencephalogram [EEG]), (7) did not provide data on individual cues (eg, only a composite score of a questionnaire), (8) were single-case studies, (9) did not focus on people with dementia, and (10) were not in English. In the case of studies using observational scales, to avoid unnecessary duplication, only the most relevant papers with unique information on cues were used, typically the original validation of each scale. No restrictions were placed on the age of publication or methodological quality.

### Charting the Data

After the 2 screening phases, 4 authors (US, SMB, AM, and MK) independently extracted data from the included articles using a standardized Microsoft Excel spreadsheet, containing several headings that correspond to our RQs, namely: reference of paper (authors, year, and DOI), type of study (eg, cross-sectional and randomized control trial), sample characteristics (total number of participants [N], target sample number and characteristics, and comparison group, if there was one, number of participants [n] and characteristics), information on dementia in the study (diagnosis and criteria or assessment), information on pain in the study (definition of pain and criteria or assessment), comorbidities (eg, other psychiatric or somatic data provided), observed cues of pain, with each cue recorded on a separate line, relation of cue to pain (direction of association), statistics provided (if available), method of observation (eg, phone, wristband, and observation), and who observed the cue (eg, caregiver or nurse).

### Collating, Summarizing, and Reporting Results

The gathered data were analyzed by 2 authors (US and SMB) using thematic analysis, in accordance with the Braun and Clarke [34] approach. This process involved categorizing closely related observable cues into higher-order categories. Several iterations were performed to refine the number of themes.

## Results

### Overview

**Figure 1.**
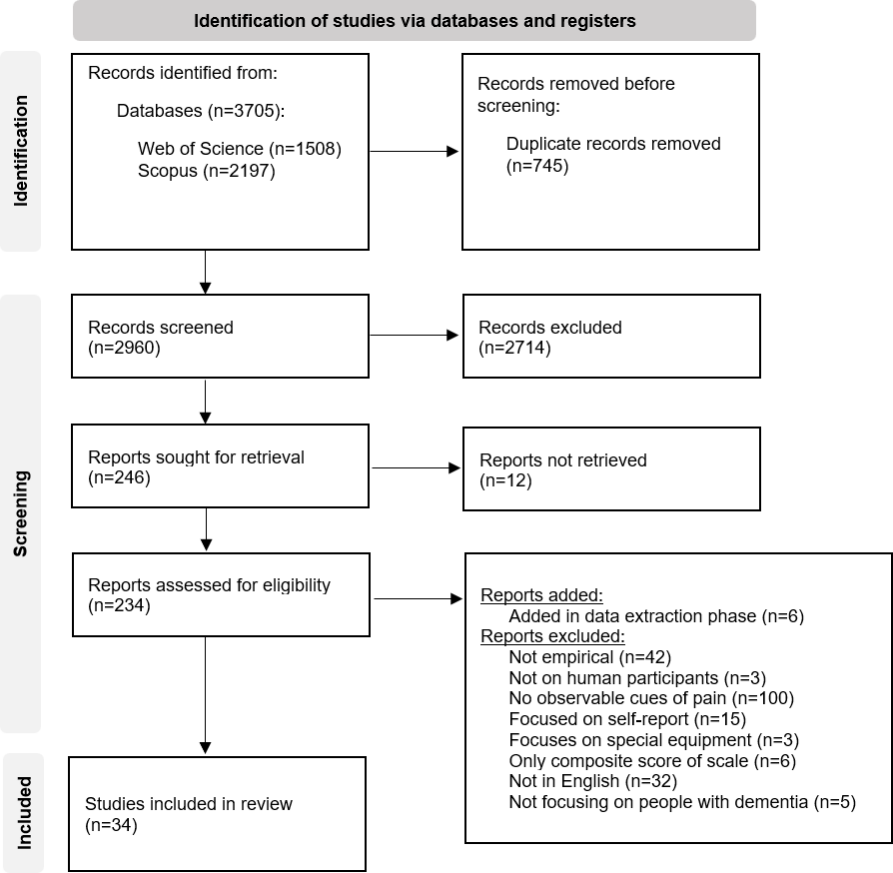
PRISMA (Preferred Reporting Items for Systematic Reviews and Meta-Analyses) flowchart depicting the study selection process.

Figure 1 shows that our search identified 3705 publications (1508 from Web of Science and 2197 from Scopus). After removing 745 duplicate records, 2960 records were screened in phase 1. The majority of those (n=2714) were excluded, leaving 246/2960 (8.3%) eligible for full-text retrieval. Additionally, 12 records were not obtainable, leaving 234/2960 (7.9%) records to be screened in phase 2. A total of 206 records were excluded based on criteria outlined in the Study Selection section. We additionally used other databases (eg, Google Scholar and PubMed) to find original validation studies for scales or questionnaires outlined in other articles, which led to the inclusion of 6 additional articles. The final sample thus consisted of 34 articles, or 1.1% of the original sample, after duplicates were removed.

Figure 1 shows that our search identified 3705 publications (1508 from Web of Science and 2197 from Scopus). After removing 745 duplicate records, 2960 records were screened in phase 1. The majority of those (n=2714) were excluded, leaving 246/2960 (8.3%) eligible for full-text retrieval. Additionally, 12 records were not obtainable, leaving 234/2960 (7.9%) records to be screened in phase 2. A total of 206 records were excluded based on criteria outlined in the previous paragraph. We additionally used other databases (eg, Google Scholar and PubMed) to find original validation studies for scales or questionnaires outlined in other articles, which led to the inclusion of 6 additional articles. The final sample thus consisted of 34 articles, or 1.1% of the original sample, after duplicates were removed.

This study analyzed 34 research articles on cues of pain in people with dementia (for details, see Table 1). Half of the studies (17/34, 50%) were published within the last decade (from 2014 onward), while most of the remaining studies (14/34, 41.2%) were published between 2004 and 2014. The largest portion (11/34, 32.4%) consisted of evaluation and validation studies. Cross-sectional studies accounted for 17.6% (6/34), while prospective observational studies and tool development studies each made up 11.8% (4/34). Most of the studies (26/34, 76.5%) did not report comorbidities in the sample or did not specify what the comorbidities were.

**Table 1.** Overview of the studies included in the review.

Reference	Study type	Total sample (Target group, comparison group)	Dementia type	Measure for identifying pain cues	Measure for validating pain cues
Atee et al [5]	Prospective observational study	40 (40)	AD^a^ (57.5%), unspecified dementia (25%), FTD^b^ (7.5%), LBD^c^ (2.5%), PD^d^ (5%), and mixed dementia (2.5%)	ePAT^e^	APS^f^
Atee et al [19]	Prospective observational study	34 (34)	AD (35.3%), unspecified dementia (44.1%), FTD (2.9%), LBD (5.9%), PD (5.9%), and VD^g^ (5.9%)	ePAT	APS
Atee et al [35]	Population-based observational retrospective study	3144 (3144)	Advanced dementia	Face domain of PainChek	—^h^
Burfield et al [36]	Longitudinal cohort study	52,996 (45,568, 7428)	CI^i^ (mild, moderate, and severe)	MDS-RAI^j^ 2.0	—
Cervo et al [37]	Development of an assessment tool	182 (182)	AD	CPAT^k^	—
Chan et al [38]	Validation study	124 (124)	CI	PACSLAC^l^ and PACSLAC-II	CNPI^m^, PADE^n^, PAINAD^o^ and NOPPAIN^p^
Cohen-Mansfield and Creedon [39]	A total of 2 interview-based studies on pain indicators	15 (15)	Not specified	Nursing staff report	—
Feldt [40]	Prospective comparative survey	88 (53, 35)	CI	CNPI	VDS^q^
Fuchs-Lacelle and Hadjistavropoulos [12]	Validation study	95 (95)	Severe CI	PASLAC	Ratings of pain between different pain events
Hodgson et al [7]	Cross-sectional study	272 (272)	Dementia (not further specified)	21 behavioral items, 16 from the ABID^r^	NIH REACH
Horgas et al [41]	Quasi-experimental correlational study	126 (62, 64)	Dementia (not further specified)	Observation of pain behaviors	self-reported pain
Hoti et al [35]	Retrospective cross-sectional study	3144 (3144)	Dementia (not further specified)	PainChek App (total pain score)	—
Husebo et al [42]	Cluster randomized controlled trial	352 (177, 177)	Dementia (not further specified)	CMAI^s^	Pain pre-post pain reducing intervention
Husebo et al [43]	Cross-sectional scale validation	352 (352, 322 [repeated measures])	Dementia (not further specified)	MOBID-2^t^	—
De Witt Jansen et al [44]	Qualitative research study	14 (14)	Advanced dementia	Interview with HCAs	—
Kaasalainen et al [45]	Tool development study	12 (12)	Not specified	Caregiver report	—
Kaasalainen et al [46]	Tool development study	14 (14)	Primary diagnosis of dementia	PACI^u^	—
Karlsson et al [47]	Exploratory qualitative study	23 (23)	Not specified	Interviews with home-care providers	VAS^v^
Kunz et al [48]	Scale validation study	685 (587, 98)	Dementia (not further specified)	Not specifically reported	—
Lints-Martindale et al [49]	Quasi-experimental study	63 (27, 36)	AD	Gracely Box Ratio Scales	Different modalities and levels of pain stimulation
Lints-Martindale et al [6]	Comparative observational study	124 (124)	Moderate to severe dementia	CAS^w^, ADD^x^, CNPI, PACSLAC, PADE, PAINAD; NOPPAIN	Different pain conditions
Lundin and Godskesen [50]	Exploratory qualitative study	13 (13)	Advanced dementia	APS, nurses report	—
Mahoney and Peters [51]	Scale validation study	112 (112)	Unspecified dementia (80%), AD (17%), FTD (2%), and VD (1%)	MPS	CMAI
Pautex et al [52]	Prospective clinical cross-sectional study	180 (131, 49)	AD (39%), mixed dementia (34%), VD (20%), or other causes (5%)	Doloplus-2	VAS
Pu et al [9]	Secondary analysis from a study	46 (46)	Dementia (not further specified)	PainChek App	—
Rezaei et al [53]	Algorithm development study	120 (48, 72)	Severe dementia	PACSLAC-II	—
Richey et al [54]	Tool development study	125 (125)	Dementia (not further specified)	PATCIE^y^	CNPI
Sandvik et al [55]	Randomized controlled trial	327 (164, 163)	AD or other dementias according to DSM	MOBID-2	Pain before/after intervention
Shega et al [56]	Prospective observational study	77 (40, 37)	Mild to moderate CI	Researchers’ observation of task	—
Takai et al [57]	Scale validation study	252 (252)	CI	APS-J^z^	Self-report
Torvik et al [58]	Cross-sectional survey	77 (77)	Severe CI	Nurses’ report	—
van de Rijt et al [59]	Cross-sectional observational study	56 (56)	Dementia (not further specified)	OPS-NVI^aa^	NRS^ab^, VDS, FPS-R^ac^
van Iersel et al [60]	Scale validation study	157 (157)	CI	Combination of items from APS and PAINAD	—
Warden et al [13]	Scale validation study	44 (44)	Advanced dementia	PAINAD	Discomfort scale and VAS scales for discomfort and pain

a AD: Alzheimer disease.

b FTD: frontotemporal dementia.

c LBD: Lewy body dementia.

d PD: Parkinson disease.

e ePAT: electronic Pain Assessment Tool.

f APS: Abbey Pain Scale.

g VD: vascular dementia.

h Not applicable.

i CI: cognitive impairment.

j MDS-RAI: Minimum Data Set Resident Assessment Instrument.

k CPAT: Computerized Pain Assessment Tool.

l PASLAC: Pain Assessment for Seniors with Limited Ability to Communicate.

m CNPI: Checklist of Nonverbal Pain Indicators.

n PADE: Pain Assessment in Dementing Elderly.

o PAINAD: Pain Assessment in Advanced Dementia.

p NOPPAIN: Non-Communicative Patient’s Pain Assessment Instrument.

q VDS: Verbal Descriptor Scale.

r ABID: Agitated Behavior in Dementia Scale.

s CMAI: Cohen-Mansfield Agitation Inventory.

t MOBID-2: Mobilization-Observation-Behavior-Intensity-Dementia-2.

u PACI: Pain Assessment Checklist for Seniors with Limited Ability to Communicate.

v VAS: visual analog scale.

w CAS: Coloured Analogue Scale.

x ADD: Assessment of Discomfort in Dementia.

y PATCIE: Pain Assessment Tool for Cognitively Impaired Elders.

z APS-J: Abbey Pain Scale - Japanese version.

aa OPS-NVI: Observational Pain Scale - Non-Verbal Individuals.

ab NRS: Numeric Rating Scale.

ac FPS-R: Faces Pain Scale - Revised.

The median sample size in the reviewed studies was 116 (range: 12-52,996). The target sample (excluding control groups) across the studies included between 12 and 45,568 participants ( mean 1537.25, SD 7581.9), predominantly older adults aged 60 years and older. Most participants (36,525/52,996, 69%) resided in long-term care facilities or nursing homes. The majority of studies (28/34, 82.4%) focused on individuals with moderate to severe dementia, including those unable to express pain. About half of the studies (18/34, 52.9%) involved participants with a history of pain-related conditions. Additionally, a subset of studies (5/34, 14.7%) involved caregivers, health care providers, or family members as study participants. About a third (11/34, 34.4%) of the included studies used comparison groups in their research design. Most of those (6/34, 54.5%) included control groups composed of cognitively intact individuals, with or without pain symptoms. Among the studies that mentioned the type of dementia (28/34, 82.4%), unspecified dementia (such as “dementia,” “advanced dementia,” and “moderate dementia”) was the most frequently mentioned (20/34, 58.8%).

A total of 211 distinct pain-related cues (485 overall instances) were extracted and categorized into 7 broad categories, each consisting of one or more subcategories. The main categories and their corresponding number of cues were: behaviors (49/211, 23.2%), body movement or expression (55/211, 26.1%), facial expressions (47/211, 22.3%), medical status–somatic (5/211, 2.4%), mental state (14/211, 6.6%), physiology (10/211, 4.7%), and speech, language, and sounds (31/211, 14.7%). The following sections describe each category in detail.

### Behaviors

We identified 5 subcategories within the “Behaviors” category: “Active behavior,” “Behavioral change,” “Inappropriate behavior,” “Mood-related behavior,” and “Social behavior” (see Table 2 for a summary, and Table S1 in Multimedia Appendix 1 for detailed information). Cues without any statistical information (ie, general behavior change) are listed in Table S8 in Multimedia Appendix 1.

**Table 2.** Summary of relevant cues of pain in the behavior category.

Pain cue			Relevance of the cue
Categories and subcategories			Direction of the association to pain^a^ and relevance of the pain cue^b^
Behavior			
Active behavior			
	General		+ (strong) [19]
	Falls		+ (weak or medium) [39]
	Impulsive behavior		+ (strong) [12]
	Normal behavior		– (strong) [37]
	Wandering		+ (strong) [5,12,54]
	Washing and/or dressing		+ (weak or medium) [52]
	Washing and/or dressing		+ (not specified)^c^ [58]
Behavioral change			
	General		+ (not specified) [51] + (strong) [57]
				Changes in appetite		+ (strong) [12,54] + (weak or medium) [39]
	Changes in communication		+ (weak or medium) [52] + (not specified) [58]			
				Changes in routines		+ (strong) [5]
	Changes in sleep		+ (strong) [5,12] + (weak or medium) [52] + (not specified) [58]
				Decrease in activity		+ (strong) [6,12] + (weak or medium) [39,52] + (not specified) [58]
							Lethargy		+ (weak or medium) [39] + (strong) [5]
	Stopping an activity		+ (weak or medium) [41]						
Inappropriate behavior			
	General		+ (strong) [5,36] + (weak or medium) [52] + (not specified) [58]
				Combativeness		+ (weak or medium) [39]
				Handling things inappropriately		+ (weak or medium) [42]
	Refusing medications		+ (strong) [12]
	Resisting care		+ (strong) [5,12,48]
	Throwing things		+ (strong) [12]
	Trying to leave or get to a different place		+ (strong) [12]
Mood-related behavior			
	Aggressive behavior		+ (strong) [5,12,54]
	Whiny		+ (weak or medium) [37]
Social behavior			
	Argumentativeness		+ (strong) [7]
	Consolability		– (strong) [13]
	Disruptive behavior		+ (strong) [54]
	Interpersonal changes		+ (strong) [6]
	Not allowing people near		+ (strong) [12,38]
	Not wanting to be touched		+ (strong) [12,38,54]
Requesting attention			+ (weak or medium) [42]
	Social life		+ (weak or medium) [52] + (weak or medium) [58]
				Striking out		+ (strong) [54]
	Unsocial behavior		+ (strong) [5]
	Withdrawn		+ (strong) [54]

a “+”=positive and “−”=negative.

b Relevance of the pain cue was determined as follows: “Strong”=large effect size and “Weak or medium”=small to medium effect size [61].

c Not specified: study reported only on the significance and direction of the association, without statistical coefficients.

In the subcategory of active behavior, general active behavior, impulsive behavior, normal behavior, and wandering were associated with pain. Falls and washing and/or dressing had mixed associations, while scratching was not significantly related to pain. Active behavior, measured using ePAT, was positively associated with APS [19]. Falls were rated as an important indicator of pain but did not show a direct relationship with pain [39,42]. Impulsive behavior, measured by PACSLAC, was associated with global pain intensity ratings [12]. Wandering, measured using the Pain Assessment Tool in Cognitively Impaired Elders (PATCIE) or PACSLAC, was linked to the Checklist of Nonverbal Pain Indicators (CNPI), APS, and global pain intensity ratings [5,12,54]. Washing or dressing behavior, measured using Doloplus-2, was associated with the Visual Analog Scale (VAS) rating [52,58].

In the behavioral change subcategory, change in communication, change in sleep, and lethargy were associated with pain, while behavioral change in general, changes in appetite or routine, decreased activity, and stopping an activity showed mixed associations. In the general behavioral change category, APS distinguished pain intensity across MMSI categories [57] and a significant difference in behavioral pain observation was found during aversive versus pleasant activities [51]. Decreased appetite was rated as important [39], and appetite changes in general, measured using PACSLAC or PATCIE, were associated with CNPI and global pain intensity ratings [12,54]. Communication changes, measured using Doloplus-2, were associated with VAS [52,58], while routine changes and sleep changes, measured using ePAT, were associated with APS [5]. Sleep changes, measured using Doloplus-2 or PACSLAC scores, were also associated with VAS or global pain ratings during events [12,52,58]. Decreased activity was rated somewhat important by nursing staff [39] and was associated with global pain intensity, as well as with Assessment of Discomfort in Dementia (ADD), CNPI, Non-Communicative Patient’s Pain Assessment Instrument (NOPPAIN), PACSLAC, Pain Assessment for the Dementing Elderly (PADE) and PAINAD, when measured using PACSLAC-II or PACSLAC [12,38,52,58]. Activity changes showed large effect sizes for both influenza vaccination and movement-exacerbated pain [6]. Lethargy was considered important by nursing staff [39], and prolonged resting, measured using ePAT, was associated with APS [5]. Pain intensity was predictive of stopping an activity pre-activity, but not during an activity [41].

In the inappropriate behavior subcategory, cues such as inappropriate behavior in general, combativeness, handling inappropriate things, refusing medication, and resisting care were associated with pain, while throwing things and trying to leave showed mixed associations. There was no association between pain and biting, eating inappropriate substances, grabbing, hiding, hitting, hoarding, hurting self or others, kicking, pushing, disrobing, spitting, or tearing things. Inappropriate behavior in general, measured using ePAT or Doloplus-2, showed a positive association with APS and VAS and predicted pain in a regression model [5,36,52,58]. Combativeness was rated as important by nursing staff [39]. Handling inappropriate things, refusing medication, resisting care, throwing things, and trying to leave were all related to pain [5,12,43,48].

In the mood-related behaviors subcategory, aggressive and whiny behavior were associated with pain, while pleasant behavior showed no association. Aggressive behavior, measured using ePAT, PATCIE, and PACSLAC, was associated with APS and CNPI [5,12,54]. Whiny behavior indicated the presence of pain [37].

In the social behavior subcategory, argumentativeness, disruptive behavior, interpersonal changes, not allowing people near, not wanting to be touched, requesting attention, social life, striking out, unsocial behavior, and being withdrawn were associated with pain. Consolability showed mixed associations, while sexual advances showed no association with pain. Argumentativeness was associated with reported pain frequency [7]. Consolability, disruptive behavior, not allowing people near, avoiding being touched, social life, striking out, unsocial behavior, and being withdrawn were associated with pain measures, such as CNPI, VAS, and APS, when measured using PAINAD, ePAT, PATCIE, PACSLAC-II, PACSLAC, or Doloplus-2 [5,12,13,38,52,54,58]. Interpersonal changes, measured using PACSLAC, showed higher effect sizes for both influenza vaccination and movement-exacerbated pain [6], while a decrease in constant need for attention was linked with decreased pain [42].

### Body Movements or Expressions

We identified 5 subcategories within the “Body movements or expressions” category: body language, body movement, body parts and cues, body positions or postures, and physical cues (see Table 3 for a summary, and Table S2 in Multimedia Appendix 1 for more detailed information). Cues without any statistical information (ie, general body language, decreased movement, reluctance to move, rubbing, tense body, and guarding) are listed in Table S8 in Multimedia Appendix 1.

**Table 3.** Summary of relevant cues of pain in the body movement or expressions category.

Pain cue			Relevance of the cue
Categories and subcategories			Direction of the association to pain^a^ and relevance of the pain cue^b^
Body movement or expressions			
Body language			
General			+ (weak or medium) [60] + (strong) [13] + (not specified) [51]
Change in body language			+ (weak or medium) [57,60]
Body movement			
General			+ (strong) [6,19]
Bracing			+ (weak or medium) [39-41] + (strong) [41]
Difficulty chewing			+ (strong) [39]
Fidgeting			+ (strong) [12]
Flinching and/or pulling away			+ (strong) [12,38]
Freezing			+ (weak or medium) [48] + (strong) [5]
Gait changes			+ (strong) [54]
Handwringing			+ (weak or medium) [37]
Leg or arm movement			+ (strong) [5]
Limping			+ (strong) [12,38,39] + (weak or medium) [39]
Pacing			+ (weak or medium) [42] + (strong) [12]
Pulling or moving away			+ (strong) [5,12]
Reluctance to move			+ (strong) [12,38,54] + (weak or medium) [39]
Repetitive movements			+ (weak or medium) [39] + (strong) [36,39]
Restlessness			+ (strong) [5,12,42,48,54,59] + (weak or medium) [7,39,59]
Rigidity			+ (weak or medium) [37,39] + (strong) [12,38,41]
Rocking motion			+ (strong) [54]
Rocking motion–head			+ (weak or medium) [39]
Rubbing			+ (mixed results) [40,56] + (strong) [38,41,48,54] + (weak or medium) [41,59]
Shaking or trembling			+ (strong) [12,38]
Shifting			+ (strong) [41] + (weak or medium) [41]
Slow movement			+ (strong) [12,38]
Tense body			+ (weak or medium) [37] + (strong) [54]
Thrashing			+ (strong) [12,38]
Tossing and/or turning			+ (weak or medium) [39] + (mixed results) [43]
Touching a body part or area			+ (strong) [12,39,54]
Trembling			+ (weak or medium) [39]
Wincing			+ (weak or medium) [39] + (strong) [39]
Body parts and cues			
General			+ (weak or medium) [36] + (strong) [19]
Abdomen			+ (mixed results) [43,55]
Arms			+ (mixed results) [43] + (strong) [55]
Hands			+ (mixed results) [43] + (weak or medium) [55]
Head, mouth, and neck			+ (mixed results) [43] + (weak or medium) [55]
Heart, lung, and chest wall			+ (weak or medium) [55] + (mixed results) [43]
Legs			+ (mixed results) [43,55]
Pelvis and genital organs			+ (mixed results) [43] + (strong) [55]
Skin			+ (mixed results) [43,55]
Turn over			+ (weak or medium) [55]
Body positions or postures			
Abnormal or awkward sitting or standing or walking			+ (strong) [5,39] + (weak or medium) [39]
Clenched fist			+ (strong) [12,38]
Fetal position			+ (strong) [12,38]
Guarding			+ (weak or medium) [41,52,56] + (strong) [5,12,38,41,48,54] + (not specified) [58]
Poor posture			+ (weak or medium) [39]
Protective posture at rest			+ (weak or medium) [52] + (not specified) [58]
Sitting			+ (mixed results) [43] + (strong) [55]
Physical cues			
Abnormal skin color			+ (strong) [39]
Blood stains			+ (weak or medium) [39]
Heat from specific body part			+ (strong) [39]
Swollen joints			+ (strong) [39]
Tight belly			+ (strong) [39]

a “+”=positive and “−”=negative.

b Relevance of the pain cue was determined as follows: “Strong”=large effect size and “Weak or medium”: small to medium effect size [61], “Mixed results”=study reported inconsistent findings, and “Not specified” =study reported only on the significance and direction of the association, without statistical coefficients.

In the body language subcategory, general body language had mixed associations, while changes in body language showed a moderate association with pain. General body language was associated with pain via behavioral pain observations, high interrater agreement, and positive association with VAS, when measured using PAINAD [13,51,60]. Changes in body language (eg, fidgeting, rocking, etc) also demonstrated high interrater agreement, as well as a connection with APS in the group exhibiting pain [57,60].

In the body movement subcategory, general body movement, and cues such as difficulty chewing, fidgeting, flinching and/or pulling away, freezing, gait changes, handwringing, leg or arm movement, limping, pulling or moving away, reluctance to move, restlessness, rocking motion, shaking or trembling, shifting, slow movement, thrashing, touching a body part, and wincing were associated with pain. Bracing, pacing, repetitive movements, rigidity, rubbing, tense body, tossing, turning, and trembling had mixed associations, while ease of movement was not associated with pain.

General movement, measured using ePAT, CNPI, NOPPAIN, PAINAD, PADE, or PACSLAC, was associated with APS or pain across conditions [6,19]. Bracing, measured using CNPI, was associated with the Verbal Descriptor Scale (VDS), linked to overall pain intensity pre- and during activity, and rated as important by nursing staff [39-41]. Difficulty chewing was rated as important by nursing staff [39], while fidgeting and flinching/pulling away, measured using PACSLAC and PACSLAC-II, showed associations with global pain ratings, CNPI, PADE, PAINAD, and NOPPAIN [12,38]. Freezing, measured using ePAT, showed an association with APS and was supported by interrater reliability [5,48]. Gait changes, assessed with PATCIE, were associated with CNPI [54]. Handwringing was associated with the presence of pain [37] and leg or arm movement, measured using ePAT, was associated with APS [5].

Limping was rated as important by nursing staff and associated with global pain ratings, NOPPAIN, CNPI, PADE, and PAINAD, when measured using PACSLAC or PACSLAC-II [12,38,39]. Pacing was associated with global pain ratings, when measured using PACSLAC, and was reduced following intervention [12,42]. Pulling or moving away, measured using ePAT or PACSLAC, was associated with global pain ratings and APS [5,12]. Reluctance to move, measured using PACSLAC, PACSLAC-II, or PATCIE, was associated with global pain intensity, CNPI, NOPPAIN, PADE, and PAINAD, and was rated as important by nursing staff [12,38,39,54].

Repetitive movements, measured using the Minimum Data Set Resident Assessment Instrument 2.0 (MDS-RAI 2.0), were linked to pain and rated as important by nursing staff [36,39]. Restlessness, measured using the caregiver report, the Cohen-Mansfield Agitation Inventory (CMAI), ePAT, PACSLAC, PATCIE, and the Observational Pain Scale – Non-Verbal Indicators (OPS-NVI), was associated with APS, CNPI, National Institutes of Health Research Evaluation and Commercialization Hubs (NIH-REACH), global pain ratings, and self-reported pain. It was also present in situations where pain was more likely and rated as important by nursing staff [5,7,12,39,43,48,54,59].

Rigidity and muscle tensing were associated with global pain intensity, NOPPAIN, CNPI, PADE, and PAINAD, when measured using PACSLAC, PACSLAC-II, or caregiver observation [12,37-39,41]. Rocking motions, assessed using PATCIE, were associated with CNPI and rated as important by nursing staff [39,54]. Rubbing, observed via caregiver observation, CNPI, OPS-NVI, or PATCIE, was associated with VDS, CNPI, or self-reported pain and was supported by interrater agreement during painful situations [40,41,48,54,59]. Shaking or trembling, trashing and slow movement, measured using PACSLAC or PACLAC-II, were associated with global pain ratings during pain events, as well as with NOPPAIN, CNPI, PADE, and PAINAD [12,38]. Shifting predicted pre-activity pain [41].

Tense body was rated as important by nursing staff and was associated with CNPI when measured using PATCIE [37,39,54]. Tossing and/or turning was also rated as important and was associated with CMAI and Neuropsychiatric Inventory—Nursing Home version (NPI-NH), when measured using Mobilization-Observation-Behavior-Intensity-Dementia-2 (MOBID-2) [39,43]. Touching a body part, measured using PACSLAC or PATCIE, was associated with global pain intensity and CNPI. It was frequently reported by nursing staff [12,39,54]. Trembling and wincing were rated as important by nursing staff [39].

The body parts and cues subcategory in general, measured using ePAT or MOBID-2, showed associations with APS, CMAI, and NPI-NH, and predicted pain in a regression model [5,36,43]. Pain in the abdomen, arms, hands, head, mouth, neck, heart, lung, chest wall, legs, pelvis, genital organs, or skin was associated with CMAI and NPI-NH, when measured using MOBID-2. Total MOBID-2 scores improved after a pain intervention [43,55].

In the body positions and postures subcategory, abnormal or awkward sitting, standing, or walking, clenched fists, fetal position, guarding, poor posture, and protective posture at rest were associated with pain, while sitting showed mixed associations. Abnormal movements or posture, measured using ePAT, were associated with APS and rated as important by nursing staff [5,39]. Clenching fists, fetal position, and guarding, measured using PACSLAC or PACSLAC-II, all had a positive association with global pain ratings, NOPPAIN, CNPI, PADE, and PAINAD [12,38]. Guarding, assessed via caregiver observation, ePAT, PATCIE, or Doloplus-2, was associated with APS, VAS, self-reported pain, or CNPI. It was supported by interrater reliability for predicting pain [5,41,48,52,54,56,58]. Poor posture was rated as important by nursing staff [39], while sitting, measured using MOBID-2, was associated with CMAI and NPI-NH [43].

Finally, Cohen-Mansfield and Creedon [39] reported that physical cues ratings by nursing staff, abnormal skin, blood stains, heat from a specific body part, swollen joints, and tight belly were all rated as important indicators of pain by nursing staff.

### Facial Expressions

We categorized facial expressions into 8 distinct subcategories: brows, cheeks, eyes, forehead, jaw, lips and mouth, nose, and whole face (see Table 4 for a summary, and Table S3 in Multimedia Appendix 1 for more detailed information). Cues without statistical information (ie, brow lowering, cheek raising, blinking, closing eyes, specific eye movement (up, down, left, or right), tightening of eyelids, jaw drop, parting lips, specific facial expression, grimacing, and sudden jerk) are listed in Table S8 in Multimedia Appendix 1.

**Table 4.** Summary of relevant cues of pain in the facial expressions category.

Pain cue			Relevance of the cue
Categories and subcategories			Direction of the association to pain^a^ and relevance of the pain cue^b^
Facial expressions			
Brows			
Brow lowering			+ (strong) [5,8,9]
Frowning			+ (weak or medium) [48] + (strong) [12,38,54,59]
Cheeks			
Cheek raising			+ (strong) [5]
Eyes			
Changes in eyes			+ (strong) [12]
Closing eyes			+ (strong) [5,8,9,38]
Dirty look			+ (strong) [12]
Increased eye movement			+ (strong) [38]
Narrowing and/or closing eyes			+ (weak or medium) [48,59] + (strong) [59]
Teary eyes			+ (strong) [12]
Tightening of eyelids			+ (strong) [5,8]
Forehead			
Creasing forehead			+ (strong) [12,38]
Jaw			
Restricting jaw movement while chewing			+ (strong) [59]
Lips and mouth			
Clenching teeth			+ (strong) [12,54]
Drooling			+ (strong) [59]
Horizontal mouth stretch			+ (strong) [5,8] + (weak or medium) [9]
Opening mouth			+ (strong) [12,38] + (weak or medium) [48,59]
Parting lips			+ (weak or medium) [8] + (strong) [5,9]
Pulling at the corner lip			+ (strong) [5] + (weak or medium) [8]
Raising of the upper lip			+ (strong) [5,8,9,48]
Nose			
Screwing up nose			+ (strong) [12]
Wrinkling nose			+ (weak or medium) [8] + (strong) [5,9]
Whole face			
General			+ (strong) [8,19]
Change in color			+ (strong) [39]
Facial expression			+ (not specified) [51,58] + (strong) [6,13,57,60]
Facial expression–specific			+ (strong) [38,60]
Fearful expression			+ (weak or medium) [37]
Flushed or red face			+ (strong) [12]
Gloomy facial expression			+ (strong) [54]
Grim face			+ (strong) [12]
Grimacing			+ (weak or medium) [39] + (mixed results) [40,56] + (strong) [12,38,41,54]
Looking tense			+ (weak or medium) [48]
Pain expression			+ (weak or medium) [52] + (strong) [12,38]
Pale face			+ (strong) [12]
Pale or flushed or red face			+ (strong) [5]
Prkachin and Solomon Pain Index			+ (weak or medium) [53]
Sad expression or look			+ (strong) [36] + (mixed results) [12]
Scared expression			+ (weak or medium) [37]
Tighter face			+ (strong) [12,38]
Wincing			+ (strong) [12,38]

a "+”=positive and “−”=negative.

b Relevance of the pain cue was determined as follows: “Strong”=large effect size, “Weak or medium”=small to medium effect size [61], “Mixed results”=study reported inconsistent findings, and “Not specified”=study reported only on the significance and direction of the association, without statistical coefficients.

The brows subcategory included brow lowering and frowning, with frowning strongly associated with pain, and brow lowering showing mixed associations. Brow lowering, measured using ePAT, was associated with APS and linked to higher pain scores in the PainChek app [5,8,9]. Frowning, measured with PACSLAC, PACSLAC-II, PATCIE, or OPS-NVI, was associated with global pain intensity, NOPPAIN, CNPI, PADE, PAINAD, and self-reported pain [12,38,48,54,59]. Cheek-raising, measured with ePAT, was associated with APS [5].

In the eyes subcategory, eye changes, closing eyes, dirty look, increased eye movement, narrowing and/or closing of eyes, and teary eyes were associated with pain, while specific eye movements (eg, looking to the left), blinking, and tightening of eyelids had mixed associations. Eye changes, measured using PACSLAC, were associated with global pain intensity [12]. Closing eyes, measured using ePAT or PainChek app, was associated with APS and observed pain and predicted pain when it was present [5,8,9]. Eye closure and increased eye movement, measured using PACSLAC-II, were associated with NOPPAIN, CNPI, PADE, and PAINAD [38]. Dirty look and teary eyes, measured using PACSLAC, were associated with global pain intensity ratings [12]. Narrowing eyes, measured using OPS-NVI and supported by interrater agreement, was associated with observational or self-reported pain [48,59]. Tightening of eyelids, measured with ePAT, was associated with APS and predicted higher pain [5,8].

In the forehead subcategory, creasing of the forehead, measured using PACSLAC or PACSLAC-II, was associated with global pain intensity, NOPPAIN, CNPI, PADE, or PAINAD [12,38]. In the jaws subcategory, restricting jaw movement was associated with self-reported pain when measured using OPS-NVI [59].

In the lips and mouth subcategory, clenching teeth, drooling, horizontal mouth stretch, opening mouth, pulling at corner of the lip, and parting lips were associated with pain, while raising of upper lip had mixed associations. Clenching teeth, measured using PACSLAC or PATCIE score, was associated with global pain intensity and CNPI [12,54]. Drooling, measured using OPS-NVI, was associated with self-reported pain [59]. Horizontal mouth stretching and parting of lips, measured using ePAT or the PainChek app, were associated with APS or observational pain scores and were more likely to predict pain when present [5,8,9].

Opening mouth, measured using interrater agreement, PACSLAC, PACSLAC-II, or OPS-NVI, was associated with self-reported pain, global pain intensity, NOPPAIN, CNPI, PADE, and PAINAD [12,38,48,59]. Pulling at the corner of lips and raising of the upper lip, measured using ePAT, were associated with APS and more likely to predict higher pain when present [5,8]. Raising the upper lip, measured using the PainChek app, OPS-NVI, or interrater agreement also had an association with observational pain scores and self-reported pain [9,48,59].

In the nose subcategory, screwing the nose and wrinkling the nose were linked with pain. Screwing the nose, measured using PACSLAC, was associated with global pain ratings [12], while wrinkling the nose, measured using ePAT or the PainChek app, was associated with APS or observational pain scores and was more likely to predict higher pain when present [5,8,9].

In the whole face subcategory, the general face domain, facial expression, fearful expression, flushed face, gloomy expression, grim face, looking tense, pain expression, pale face, Prkachin and Solomon Pain Intensity, scared expression, tight face, and wincing were associated with pain, while change in color, grimacing, sad expression, and sudden jerks had mixed associations. A relaxed expression had no association with pain. The face domain, measured using ePAT, was associated with APS. Whole-face domain scores were significantly associated with pain, with upper face action units (eg, brows, eyelids, and eyes) noted more frequently than lower face action units (eg, nose and lips) during moderate and severe pain [8,19]. Change in face color was rated as important by nursing staff [39].

Facial expressions measured using APS, Coloured Analogue Scale (CAS), ADD, CNPI, Mahoney Pain Scale (MPS), PACSLAC, PADE, PAINAD, and NOPPAIN differentiated pain conditions and levels of dementia. When measured using Doloplus-2, PAINAD, or PACSLAC-II, facial expressions were associated with caregiver-reported pain, VAS, NOPPAIN, CNPI, PADE, and PAINAD. Facial expressions were also rated as reliable indicators of pain by interrater agreement [6,13,38,51,57,58,60]. Specifically, brow lowering, lid tightening, cheek raising, and jaw clenching, looking tense, frowning, grimacing, or appearing frightened were identified as pain indicators based on caregiver report or interrater agreement [60]. A fearful expression had a significant association with the presence of pain [37]. Flushed, red face and grim face, measured using PACSLAC, were associated with global pain ratings [12], while gloomy facial expression, measured using PATCIE, was associated with CNPI [54]. Grimacing, measured using PACSLAC, PACSLAC-II, PATCIE CNPI, or observation, was associated with global pain rating, self-rated pain, VDS, NOPPAIN, CNPI, PADE, or PAINAD. It was rated as important by nursing staff and was more frequent in patients with chronic low back pain than in pain-free participants [12,38-41,54,56]. Looking tense was present in situations where pain was more likely, based on interrater agreement or intraclass correlation coefficient [48]. Pain expression, measured using PACSLAC, PACSLAC-II, or Doloplus-2, was associated with global pain intensity, NOPPAIN, CNPI, PADE, and PAINAD [12,38,52]. Pale face, measured using PACSLAC, was associated with global pain intensity [12], while pale and/or flushed (red) face, measured using ePAT, was associated with APS [5].

Prkachin and Solomon pain estimation model outperformed a baseline model in pain estimation [53]. Scared expression, measured using Computerized Pain Assessment Tool (CPAT), was associated with the caregiver’s report of pain [37], while sad expression predicted pain in a regression model and was associated with pain intensity when measured using PACSLAC [12,36]. Tighter face and wincing, measured using PACSLAC or PACSLAC-II, were associated with NOPPAIN, CNPI, PADE, PAINAD, and global pain intensity [12,38].

### Medical Status: Somatic

The “Medical status—somatic” category included 2 subcategories, injuries and medical conditions (see Table 5 for a summary, and Table S4 in Multimedia Appendix 1 for more details).

**Table 5.** Summary of relevant cues of pain in the medical status—somatic category.

Pain cue			Relevance of the cue		
Category and subcategories			Direction of the association to pain^a^ and relevance of the pain cue^b^		
Medical status–somatic					
Injuries					
Dislocated limbs			+ (strong) [39]		
Injuries			+ (strong) [5]		
Physical changes			+ (weak or medium) [60]		
Medical conditions					
One leg shorter			+ (weak or medium) [39]		
Painful medical conditions			+ (strong) [5] + (mixed results) [51]		

a "+”=positive and “−”=negative.

b Relevance of the pain cue was determined as follows: “Strong”=large effect size, “Weak or medium”=small to medium effect size [61], and “Mixed results”=study reported inconsistent findings.

In this category, all cues were associated with pain, except for painful medical conditions, which had mixed associations. Dislocated limbs and having one leg shorter were rated as important by nursing staff [39]. Injuries in general, and painful medical conditions, when measured using ePAT, were associated with APS [5], but the latter showed no connection to pain, when measured using MPS [51]. Physical changes such as skin tears, pressure areas, and arthritis were reported as good indicators of pain based on high inter-rater agreement [60].

### Mental State or Mood

The mental state or mood category had 2 subcategories: mental states and mood indicators (see Table 6 for a summary, and Table S5 in Multimedia Appendix 1 for more details). Cues that were reported without any statistical information (ie, depression) can be found in Table S8 in Multimedia Appendix 1.

**Table 6.** Summary of relevant cues of pain in the mental state and mood categories.

Pain cue			Relevance of the cue
Category and subcategories			Direction of the association to pain^a^ and relevance of the pain cue^b^
Mental state or mood			
Mental state			
Changes in mental status			+ (strong) [6,38,39]
Confusion			+ (strong) [5,12,54]
Delusions			+ (weak or medium) [7]
Distressed			+ (strong) [5]
Mood indicators			
Agitation			+ (weak or medium) [7,39]
			+ (strong) [12]
Anger			+ (strong) [12]
Anxiousness, nervousness			+ (weak or medium) [7,12] + (strong) [7,12]
Depression			+ (weak or medium) [39]
Fear			+ (strong) [5]
Frustrated			+ (strong) [12]
Irritable			+ (strong) [12,39,54]
Mood changes			+ (weak or medium) [36]
Moodiness			+ (weak or medium) [39] + (strong) [12]

a Direction of the association to pain: “+”=positive and “−”=negative.

b Relevance of the pain cue was determined as follows: “Strong”=large effect size and “Weak or medium”=small to medium effect size [61].

Mental state cues, such as changes in mental status, confusion, delusions, and distress, were associated with pain. Changes in mental status, when measured using PACSLAC, ADD, CAS, CNPI, PADE, PAINAD, and NOPPAIN, differentiated between pain conditions. When measured with PACSLAC-II, mental status changes were also associated with NOPPAIN, CNPI, PADE, and PAINAD [6,38]. Increased agitation, moodiness, and irritability were frequently reported by nursing staff [39]. Confusion, measured using ePAT, PATCIE, or PACSLAC, was associated with APS, CNPI, and global pain ratings [5,12,54]. Delusions and distress, measured using ePAT or behavioral report items, were associated with APS and NIH-REACH [5,7].

Mood indicators, such as agitation, anger, anxiety, depression, fear, frustration, irritability, mood changes, and moodiness were associated with pain. Agitation was rated as important by nursing staff and was associated with NIH-REACH and global pain intensity, measured using items on behavioral report and PACSLAC [7,12,39]. Both anger and anxiety, measured using PACSLAC, were associated with global pain intensity [12]. Anxiety, measured by behavioral items, was associated with NIH-REACH [7].

Depression was rated as important by nursing staff [39]. Fear, measured using ePAT, was associated with APS [5], while frustration and irritability, measured using PACSLAC, were associated with global pain intensity [12]. Irritability was associated with CNPI when measured using PATCIE and was rated as important by nursing staff [39,54]. Mood changes, measured with MDS-RAI 2.0, were moderately connected to pain in a regression model [36]. Moodiness, measured using PACSLAC, was associated with global pain intensity and rated as important by nursing staff [12,39].

### Physiology

In the “Physiology” category, we identified 4 subcategories: body temperature, breathing, other indicators, and several vital signs (see Table 7 for a summary, and Table S6 in Multimedia Appendix 1 for more details). Cues without statistical information (ie, general changes in vital signs) can be found in Table S8 in Multimedia Appendix 1.

**Table 7.** Summary of relevant cues of pain in the physiology category.

Pain cue			Relevance of the cue
Category and subcategories			Direction of the association to pain^a^ and relevance of the pain cue^b^
Physiology			
Body temperature			
Cold or feverish			+ (strong) [5]
Cold and clammy			+ (strong) [12]
Sweating			+ (strong) [5,12,39]
Breathing			
Ease of breathing			– (not specified) [51] – (strong) [13]
Gasping or breathing loudly			+ (weak or medium) [39] + (strong) [38]
Rapid breathing			+ (strong) [5]
Other			
Physical change			+ (weak or medium) [57]
Vomiting			+ (weak or medium) [39]
Vital signs–several			
Changes in vital signs–general			+ (strong) [39,57]
			+ (mixed results) [51]

a Direction of the association to pain: “+”=positive and “–”=negative.

b Relevance of the pain cue was determined as follows: “Strong”=large effect size, “Weak or medium”=small to medium effect size [61], “Mixed results”=study reported inconsistent findings, and “Not specified”=study reported only on the significance and direction of the association, without statistical coefficients.

Within the body temperature subcategory, being cold, clammy, feverish, and/or sweating, measured using ePAT or PACSLAC, was associated with global pain intensity and APS. Sweating was also rated as important by nursing staff [5,12,39].

Within the breathing subcategory, ease of breathing, measured using MPS or PAINAD, differentiated aversive and pleasant activities and was associated with VAS [13,51]. Gasping or breathing loudly, measured using PACSLAC-II, was associated with NOPPAIN, CNPI, PADE, and PAINAD and was rated as important by nursing staff [38,39]. Rapid breathing measured using ePAT was associated with APS [5].

In the other indicators subcategory, vomiting was rated as important by nursing staff [39], while physical changes, measured using APS, were associated with self-reported pain and differentiated pain and dementia groups [57].

For the several vital signs subcategory, associations with pain were mixed. General changes in vital signs were rated as important by nursing staff. When measured using MPS or APS, they were associated with functional deviations and self-reported pain [39,51,57].

### Speech, Language, and Sounds

In the speech, language, and sounds category, 7 subcategories were identified: complaining, inappropriate verbal behavior, language, nonverbal expression, speech, verbal expression, and other (see Table 8 for summary, and Table S7 in Multimedia Appendix 1 for more details). Cues without any statistical information (ie, nonverbal expression in general and negative vocalizations) are listed in Table S8 in Multimedia Appendix 1.

**Table 8.** Summary of relevant cues of pain in the speech, language, and sounds categories.

Pain cue	Relevance of the cue
Category and subcategories	Direction of the association to pain^a^ and relevance of the pain cue^b^
Speech, language, and sounds	
Other	
General	+ (strong) [19]
Pain frequency	+ (weak or medium) [36]
Pain intensity	+ (weak or medium) [36]
Quietness	+ (weak or medium) [39]
Complaining	
Somatic complaints	+ (weak or medium) [52] + (not specified) [58]
Verbal complaints	+ (strong) [41,42,48] + (mixed results) [40]
Inappropriate verbal behavior	
Verbal aggression	+ (weak or medium) [42] + (strong) [12]
Verbally offensive	+ (strong) [5]
Language	
Calling out	+ (strong) [12] + (weak or medium) [37]
Negative statements	+ (weak or medium) [36]
Negativism	+ (strong) [42]
Repetitive verbalizations	+ (weak or medium) [36,42]
Requesting help repeatedly	+ (strong) [5,35]
Nonverbal expression	
General	+ (weak or medium) [57] + (mixed results) [40] + (strong) [6]
Crying	+ (strong) [5,12,35,36,38,54] + (weak or medium) [39]
Groaning	+ (strong) [5,35,48,54]
Grunting	+ (strong) [12,38,54]
Howling	+ (strong) [5,35]
Moaning	+ (strong) [5,35,37,54]
Moaning and groaning	+ (strong) [12,38]
Mumbling	+ (strong) [12,48]
Negative vocalizations	+ (weak or medium) [39,60] + (strong) [13,46] + (not specified) [51]
Screaming	+ (weak or medium) [39] + (strong) [5,12,35]
Shouting	+ (strong) [48]
Sighing	+ (strong) [5,35,41,54]
Whining	+ (strong) [54]
Yelling	+ (strong) [54]
Speech	
Loud talk	+ (strong) [5,35]
Verbal expression	
Specific pain sounds or words	+ (strong) [5,12,35,38,46,48]

a Direction of the association to pain: “+”=positive and “−”=negative.

b Relevance of the pain cue was determined as follows: “Strong”=large effect size, “Weak or medium”=small to medium effect size [61], “Mixed results”=study reported inconsistent findings, and “Not specified”=study reported only on the significance and direction of the association, without statistical coefficients.

In the complaining subcategory, somatic complaints, measured using Doloplus-2, were associated with pain [52,58]. Verbal complaints, measured using CNPI, observation, or interrater agreement, were associated with pain, as measured with VDS or self-report. Verbal complaints significantly decreased after a pain-reducing intervention [40,41,43,48].

In the inappropriate verbal behavior subcategory, verbal aggression and verbally offensive behavior were associated with pain, while verbal sexual offenses showed no association. Verbal aggression and offensive behavior, measured using PACSLAC, CMAI, or ePAT, were associated with pain intensity, APS, and were reduced following a pain-reducing intervention [5,12,42].

In the language subcategory, both negative statements or negativism and repetitive verbalizations predicted pain in a regression model and were reduced after pain treatment [36,42].

In the nonverbal expression subcategory, crying, groaning, grunting, howling, moaning, mumbling, negative vocalizations, shouting, whining, and yelling were associated with pain. Vocalizations and screaming had mixed associations, while making strange noises had no association with pain.

Nonverbal expressions in general, measured using APS, CNPI, CAS, ADD, PACSLAC, PADE, PAINAD, or NOPPAIN, were associated with VDS and self-reported pain and distinguished between different pain conditions [6,40,57]. Crying and groaning, measured using ePAT, MDS-RAI 2.0, PACSLAC, PACSLAC-II, or PATCIE, were associated with global pain intensity, APS, NOPPAIN, CNPI, PADE, or PAINAD. They were rated as important by nursing staff and had a high effect size for predicting pain [5,12,35,36,38,39,54]. Grunting, measured using PACSLAC, PACSLAC-II, or PATCIE, was associated with global pain intensity, NOPPAIN, CNPI, PADE, or PAINAD [12,38,54].

Howling, measured using ePAT, was associated with APS and had a high effect size in predicting pain [5,35]. Moaning, measured using ePAT, PATCIE, or CPAT, was associated with APS, CNPI, and general pain presence. It also had a high effect size in predicting pain [5,35,37,54]. Combined with groaning and mumbling, it was associated with NOPPAIN, CNPI, PADE, and PAINAD, or global pain intensity, when measured using PACSLAC or PACSLAC-II [12,38]. Mumbling was present in pain-like situations, supported by interrater agreement [48].

Negative vocalizations had high interrater agreement as pain indicators, were rated as important by nursing staff, and were associated with pain behaviors across conditions, and with VAS, when measured with MPS, the Pain Assessment Checklist for Seniors with Limited Ability to Communicate (PACI) or PAINAD [13,39,46,51,60]. Screaming, measured using ePAT or PACSLAC, was associated with global pain intensity or APS. It was rated as important by nursing staff and had a large effect size in predicting pain scores when present [5,12,35,39].

Shouting was present in pain-like situations, as rated by interrater agreement [48], while sighing, measured using ePAT or PATCIE, was associated with APS or CNPI. It had a large effect size for predicting pain and pain behaviors in general [5,35,41,54]. Whining and yelling, measured using PATCIE, were associated with CNPI [54].

In the speech subcategory, loud talk, measured using ePAT, was associated with APS and predicted pain scores when pain was present with a large effect size [5,35]. Calling out, measured using CPAT or PACSLAC, was associated with pain presence or global pain intensity [12,37].

In the verbal expression subcategory, specific pain sounds or words (eg, “ow” and “ouch”) and requesting help repeatedly, measured using ePAT, were associated with APS and had a large effect size in predicting pain scores when present [5,35]. Additionally, pain sounds measured using PACI, PACSLAC, or PACSLAC-II were associated with global pain intensity, caregiver report, NOPPAIN, CNPI, PADE, or PAINAD. They were also present in situations where pain was more likely, as supported by interrater agreement [12,38,46,48].

In the “other” subcategory, the voice domain, measured using ePAT, was associated with APS [19]. Pain intensity and frequency, as described or displayed by the patient, showed moderate effects in regression models [36], and unusual silence was rated as important by nursing staff [39].

### Methods of Measuring Pain Cues

Most cues were assessed through human observation, either directly or from recorded footage. A total of 2 studies combined human observation with facial recognition software [8,19], another used both human observation and the PainChek App on a tablet [9], and 1 study reported integrating computer vision with human observation [53].

## Discussion

### Principal Findings

Individuals with dementia often have difficulties or are unable to self-report the experience of pain [8], which frequently results in its underassessment, especially for patients with moderate to severe dementia [5,6]. Recognition and assessment of pain could significantly improve with the use of digital monitoring, which would help identify pain cues in patients with (advanced) dementia [5,8,19,20]. This review aimed to identify such observable cues that could be used to detect pain in patients with dementia by way of digital monitoring. More specifically, we wanted to examine, “which digital cues offered a valid insight into pain in people with dementia” (RQ1) and identify “how these cues were originally measured” (RQ2).

A total of 34 relevant articles on observable cues of pain were closely examined in this scoping review. Addressing the first RQ, we could identify several behavioral and physiological cues associated with pain, which were categorized into 7 main categories. For each of the main categories, several subcategories of pain cues were identified, each involving between 1 and 28 specific pain cues. In the category of behavior, observing wandering [5,12,54], changes in appetite [12,54], inappropriate [5,36], aggressive [5,12,54], and disruptive behavior [54], as well as not wanting to be touched [12,38,54], was most strongly associated with the experience of pain, according to the available data. Considering body movements and expressions, the most relevant cues for identifying pain were flinching and/or pulling away [12,38], shaking or trembling, clenching one’s fists or adopting a fetal position [12,38], and swollen joints [38,39]. In the category of facial expressions, lowering the brows [5,8,9], closing the eyes [5,8,9,38], creasing the forehead [12,38], clenching one’s teeth [12,54], or raising one’s upper lip [5,8,9,48], as well as wincing [12,38], were most relevant for pain identification. Further identified observable pain cues were dislocated limbs [39] and injuries [5], falling into the category of medical or somatic pain cues. Within the category of mental state and mood, changes in mental status [6,38,39], being confused [5,12,54], and distressed [5] or irritable [12,39,54] were the most important cues for identifying pain. Within physiology-related cues, being cold or feverish [5] or cold and clammy [12], sweating [5,12,39], or breathing rapidly [5], strongly signified the experience of pain. Finally, considering speech, language, and sounds, the most relevant pain cues were being verbally offensive [5], requesting help repeatedly [5,35], groaning [5,35,48,54], moaning [5,35,37,54], talking loudly [5,35], and using specific pain sounds or words [5,12,35,38,46,48].

Individuals with dementia often experience pain but may not know how to express it or may be unable to do so. Several methods of observing pain exist, but they often lack objectivity, differ in reliability and validity—leading to inconsistent assessments—or focus only on certain cues, thereby failing to account for all relevant ones, mainly due to time constraints and the effort required for a comprehensive assessment [5,6,14,15]. This review focused on recognizing various observable pain cues, and the most relevant cues were identified. They align with and extend previous frameworks, such as the American Geriatrics Society guidelines, which categorize nonverbal pain cues to be considered when identifying pain in people with dementia into facial expressions, verbalizations, vocalizations, body movements, changes in interpersonal interactions, in activity patterns or routines, and in mental status [22,62].

Findings of this review can serve as a basis for an objective identification of pain, especially in individuals with moderate or severe dementia. Even though several identified cues correspond to, for instance, already established pain-related facial action units identified in nondementia populations, for example, in the study by Prkachin and Solomon [63], pain expression can potentially be altered in people with dementia due to impaired cognitive and communicative abilities [64]. Therefore, focused, in-depth investigations of pain cues in people with dementia are of utmost importance.

While several observable cues of pain in individuals with dementia were identified, further investigation into this topic is necessary, as support for some cues is inconsistent. Specifically, the strength of pain associations with some cues varied between strong and weak or medium, or mixed results. A potential explanation of inconsistent results lies in the suggestion of previous studies regarding the variability in pain expressions, which depend on the contextual influences (eg, pain type), dementia type, and its severity [65], as well as the potential of some cues being misattributed to psychiatric causes rather than pain (eg, behavioral cues like wandering and aggression that overlap with agitation symptoms in dementia) [66]. These reasons deem the in-depth exploration of the topic even more important.

Some reviewed studies also failed to report the strength or statistical significance of the reported associations. Additionally, many of the cues were quite broadly defined or lacked a detailed description, hindering insight into the specifics of certain observational cues.

Addressing the second RQ, most of the reported pain cues were assessed through human observation, although some studies also included a facial recognition software [8,19], the PainChek app [9,20], or combined human observation with computer vision [53]. Human observation certainly improves reliability, especially when patients are not able to express pain themselves, as shown by the very validation studies we examined for relevant pain cues (eg, [12,13,38]). Nevertheless, human observation introduces a certain amount of bias and, more importantly, requires significant time and effort. It is also important to point out that a large proportion of the included papers consisted of validation studies of specific (observational) questionnaires. Although these questionnaires were designed for the purpose of others (eg, caregivers or nurses) assessing levels of pain in individuals with dementia, such studies do not provide information on how such cues could be digitally monitored—an important issue for future studies or efforts for digital pain identification. Nevertheless, a few of the identified pain cues were assessed with facial recognition software or computer vision. A prominent example of facial recognition, the PainChek app, uses automated facial recognition and analysis to detect facial action units associated with pain. It also incorporates user-fed clinical data to generate a pain intensity score. Additionally, the app offers various features, including pain assessment, monitoring, patient profiling, and data synchronization, although these require active human participation and human cue observation [20]. As such, the PainChek app represents a solution that could potentially be further developed with the use of findings from this paper, with the ultimate goal of automatic digital pain detection. Specifically, automatic recognition of pain cues could be extended beyond the use of only facial action units to include a broader scope of cues that were identified in this review. Incorporating such multimodal approaches, relying on various types of pain cues for its detection, AI systems could provide greater consistency in pain recognition and potential for real-time monitoring, enhancing the diagnostic accuracy and accessibility of reliable pain assessment in people with dementia. In the AI4HOPE [24] project, we intend to exploit the findings of this review for the development of explainable AI able to capture and interpret cues of pain that are spontaneously expressed.

### Implications

To the best of our knowledge, this review serves as a first overview of cues for the purpose of digitally monitoring cues related to pain. To utilize these cues in pain assessment, it is necessary to explore ways to design efficient algorithms and to investigate which cues are better or worse predictors of pain. The findings of this paper show that potential cues of pain exist in many different categories (eg, behavior, body movements, facial expressions, and language). This is particularly important for the development of algorithms for the purpose of identifying or classifying the identified cues of pain, as it has been found that algorithms, which consider multiple modalities, tend to be more successful than those focusing on a single modality in identifying other conditions, for example, depression [67,68]. The identified pain cues could also be used to develop digital solutions that are on the rise for supporting people with dementia [69], for example, based on AI algorithms, to help identify pain in individuals with dementia. AI-based systems could offer a more efficient and cost-effective method for clinicians to assess pain. These solutions would also allow for continuous, real-time pain monitoring, which is not feasible with traditional observational or self-reported measures. For individuals with dementia, AI would provide a discreet and accessible way to monitor pain. It would benefit clinicians by not requiring much conscious effort and allowing for continuous assessments, as well as help patients by reducing the reliance on self-reporting, which can be especially challenging for those unable to communicate their pain. Additionally, such solutions could be incorporated into broader platforms collecting various disease-related cues over longer periods of time, benefiting researchers and clinicians in gaining insight into disease progression, in order to improve prevention strategies, interventions, and personalized care for patients with dementia (eg, [70]).

### Limitations

This scoping review provides a valuable synthesis of research on the observable cues of pain in individuals with dementia and their measurement. However, certain limitations must be acknowledged. Since we focused exclusively on English-language publications, predominantly involving Anglophone participants from industrialized nations, our findings may not be fully generalizable across different cultural contexts. Another limitation lies in the fact that the largest proportion of the included papers consisted of evaluation and validation studies, which may have led to a narrower focus, as these studies concentrate on the effectiveness or reliability of specific interventions or tools, but do not address all relevant aspects of the phenomenon in question. This limitation of the study distribution may have resulted in a less comprehensive understanding of the topic, particularly in terms of causality and breadth of applications. However, it is important to note that there is a general lack of studies that evaluate observable cues indicative of pain, which is why such a large proportion of evaluation and validation studies were used in this review. Finally, as a scoping review using established methodology [25] was conducted to provide a comprehensive overview of the research related to our RQs, the quality of included articles was not assessed. Future research could additionally perform a risk of bias assessment.

### Conclusions

This review examined the relevant literature for the purpose of identifying observable cues that could be used to detect pain in people with dementia with the use of digital monitoring. We focused on cues that can be measured without the use of specialized equipment unavailable to the general public. The review resulted in a comprehensive set of observable cues that could be used to help identify pain in persons with dementia. We also identified inconsistencies regarding the relevance of some identified cues, as well as a lack of studies that evaluate observable cues indicative of pain, beyond the scope of evaluation and validation studies of specific pain questionnaires. The conducted review could help future advancements aiming to objectively and efficiently identify and manage pain in persons with dementia, potentially via the use of AI and digital monitoring. Our findings could also inform the design of accessible devices (eg, smartwatches and smartphones), to incorporate features that monitor and assess pain.

## Supplementary material

10.2196/75671 Multimedia Appendix 1 Summaries of relevant and irrelevant cues of pain in specific subcategories.

10.2196/75671 Checklist 1 PRISMA-ScR checklist.

## Abbreviations

ADD: Assessment of Discomfort in Dementia
AI: artificial intelligence
APS: Abbey Pain Scale
CAS: Coloured Analogue Scale
CMAI: Cohen-Mansfield Agitation Inventory
CNPI: Checklist of Nonverbal Pain Indicators
CPAT: Computerized Pain Assessment Tool
ePAT: electronic Pain Assessment Tool
MDS-RAI: Minimum Data Set Resident Assessment Instrument
MOBID-2: Mobilization-Observation-Behavior-Intensity-Dementia-2
NIH-REACH: National Institutes of Health Research Evaluation and Commercialization Hubs
NOPPAIN: Non-Communicative Patient's Pain Assessment Instrument
NPI-NH: Neuropsychiatric Inventory—Nursing Home version
OPS-NVI: Observational Pain Scale – Non-Verbal Individuals
PACI: Pain Assessment Checklist for Seniors with Limited Ability to Communicate
PACSLAC: Pain Assessment Checklist for Seniors with Severe Dementia
PADE: Pain Assessment in Dementing Elderly
PAINAD: Pain Assessment in Advanced Dementia
PATCIE: Pain Assessment Tool in Cognitively Impaired Elders
PRISMA-ScR: Preferred Reporting Items for Systematic Reviews and Meta-Analyses Extension for Scoping Reviews
RQ: research question
VDS: Verbal Descriptor Scale
