# Supplementary material for: Pain Cues in People With Dementia: Scoping Review
Source: JMIR Ment Health. 2025 Nov 27;12:e75671. doi: 10.2196/75671 (PMC12661616; doi:10.2196/75671)
Supplement: Multimedia Appendix 1 [file mental-v12-e75671-s001.pdf]

Supplementary Materials

Decoding Pain: A Scoping Review of Pain Cues in People with Dementia

Urška Smrke*, Ana Milošič, Izidor Mlakar, Matic Kadiš, Satja Mulej Bratec

*Correspondence: urska.smrke@um.si

Table S1. *Summary of relevant cues of pain in the "behavior" subcategory.*

Table S2. *Summary of relevant cues of pain in the "body movement/expressions" subcategory*.

Table S3. *Summary of relevant cues of pain in the "facial expressions" subcategory*.

Table S4. *Summary of relevant cues of pain in the "medical status - somatic" subcategory*.

Table S5. *Summary of relevant cues of pain in the "mental state / mood" subcategory.*

Table S6. *Summary of relevant cues of pain in the "physiology" subcategory.*

Table S7. *Summary of relevant cues of pain in the "speech, language and sounds" subcategory.*

Table S8. *Summary of irrelevant cues or cues with no reported statistical information across categories.*

Table S1
Summary of relevant cues of pain in the "behavior" subcategory.

Pain cue			Relevance of the cue		Study	
**Category 1**	**Category 2**	**Category 3**	**Relevance and association direction of pain cue**	**Description of association**	**Authors**	**Year**
Behavior	Active behavior	[General]	Strong, +	Association of the ePAT and APS scales	Atee et al.	2018
		Falls	Weak/medium, +	Marker importance as rated by the nursing staff	Cohen-Mansfield & Creedon	2002
		Impulsive behavior	Strong, +	Association of the PACSLAC scale and global pain intensity rating during pain events	Fuchs-Lacelle & Hadjistavropoulos	2004
		Normal behavior	Strong, +	Significant association for the absence of pain	Cervo et al.	2007
		Wandering	Strong, +	Association of the ePAT and APS scales	Atee et al.	2017
			Strong, +	Association of the PACSLAC scale and global pain intensity rating during pain events	Fuchs-Lacelle & Hadjistavropoulos	2004
			Strong, +	Association of the CNPI and PATCIE scales in relation to movement	Richey et al.	2020
		Washing &/or dressing	Weak/medium, +	Association of the Doloplus-2 with self-reported pain (VAS)	Pautex et al.	2007
			Not specified, +	More patients evaluated as experiencing pain with Doloplus-2 than with a single question	Torvik et al.	2010
	Behavioral change	[General]	Not specified, +	More behavioral pain observations during aversive than pleasant activity for pain and agitation groups in relation to controls	Mahoney & Peters	2008
			Strong, +	Association between Abbey pain scale and self-report	Takai et al.	2014
		Changes in appetite	Strong, +	Association of the CNPI and PATCIE scales in relation to movement	Richey et al.	2020
			Strong, +	Association of the PACSLAC scale and global pain intensity rating during pain events	Fuchs-Lacelle & Hadjistavropoulos	2004
			Weak/medium, +	Marker importance as rated by the nursing staff	Cohen-Mansfield & Creedon	2002
		Changes in communication	Weak/medium, +	Association of the Doloplus-2 with self-reported pain (VAS)	Pautex et al.	2007
			Not specified, +	More patients evaluated as experiencing pain with Doloplus-2 than with a single question	Torvik et al.	2010
		Changes in routines	Strong, +	Association of the ePAT and APS scales	Atee et al.	2017
		Changes in sleep	Strong, +	Association of the ePAT and APS scales	Atee et al.	2017
			Strong, +	Association of the PACSLAC scale and global pain intensity rating during pain events	Fuchs-Lacelle & Hadjistavropoulos	2004
			Weak/medium, +	Association of the Doloplus-2 with self-reported pain (VAS)	Pautex et al.	2007
			Not specified, +	More patients evaluated as experiencing pain with Doloplus-2 than with a single question	Torvik et al.	2010

	Decrease in activity	Strong, +	Higher PACSLAC score during pain events. Other tools investigated all had adequate psychometric properties and distinguished painful and non-painful states	Lints-Martindale et al.	2012
		Strong, +	Association of the PACSLAC scale and global pain intensity rating during pain events	Fuchs-Lacelle & Hadjistavropoulos	2004
		Strong, +	Association of a global PASLAC-II score with other pain assessments (PASLAC, NOPPAIN, CNPI, PADE, PAINAD)	Chan et al.	2014
		Weak/medium, +	Marker importance as rated by the nursing staff	Cohen-Mansfield & Creedon	2002
		Weak/medium, +	Association of the Doloplus-2 with self-reported pain (VAS)	Pautex et al.	2007
		Not specified, +	More patients evaluated as experiencing pain with Doloplus-2 than with a single question	Torvik et al.	2010
	Lethargy	Weak/medium, +	Marker importance as rated by the nursing staff	Cohen-Mansfield & Creedon	2002
		Strong, +	Association of the ePAT and APS scales	Atee et al.	2017
	Stopping an activity	Weak/medium, +	Pain intensity was predictive of the behaviour	Horgas et al.	2009
Inappropriate behavior	[General]	Strong, +	Association of the ePAT and APS scales	Atee et al.	2017
		Strong, +	Marker rated as important in a recursive model	Burfield et al.	2012
		Weak/medium, +	Association of the Doloplus-2 with self-reported pain (VAS)	Pautex et al.	2007
		Weak/medium, +	Association of the shortened Doloplus-2 with self-reported pain (VAS)	Pautex et al.	2007
		Not specified, +	More patients evaluated as experiencing pain with Doloplus-2 than with a single question	Torvik et al.	2010
	Combativeness	Weak/medium, +	Marker importance as rated by the nursing staff	Cohen-Mansfield & Creedon	2002
	Handling things inappropriate	Weak/medium, +	significant decrease from baseline to 8 weeks between control and intervention group (i.e., linked to decreased pain)	Husebo, Ballard et al.	2013
	Refusing medications	Strong, +	Association of the PACSLAC scale and global pain intensity rating during pain events	Fuchs-Lacelle & Hadjistavropoulos	2004
	Resisting care	Strong, +	Marker present when pain was more likely, as rated by sufficient interrater agreement or intraclass correlation coefficient	Kunz et al.	2019
		Strong, +	Association of the ePAT and APS scales	Atee et al.	2017
		Strong, +	Association of the PACSLAC scale and global pain intensity rating during pain events	Fuchs-Lacelle & Hadjistavropoulos	2004
	Throwing things	Strong, +	Association of the PACSLAC scale and global pain intensity rating during pain events	Fuchs-Lacelle & Hadjistavropoulos	2004
	Trying to leave/get to a different place	Strong, +	Association of the PACSLAC scale and global pain intensity rating during pain events	Fuchs-Lacelle & Hadjistavropoulos	2004
Mood-related behaviour	Aggressive behavior	Strong, +	Association of the ePAT and APS scales	Atee et al.	2017
		Strong, +	Association of the CNPI and PATCIE scales in relation to movement	Richey et al.	2020

		Strong, +	Association of the PACSLAC scale and global pain intensity rating during pain events	Fuchs-Lacelle & Hadjistavropoulos	2004
	Whiny	Weak/medium, +	Marker presence associated with presence of pain	Cervo et al.	2007
Social behavior	Argumentativeness	Strong, +	Significant relationship between the degree of pain and the frequency of BPSD	Hodgson et al.	2014
	Consolability	Strong, +	Association of total PAINAD and VAS pain scale	Warden et al.	2003
	Disruptive behavior	Strong, +	Association of the CNPI and PATCIE scales in relation to movement	Richey et al.	2020
	Interpersonal changes	Strong, +	Higher PACSLAC score during pain events. Other tools investigated all had adequate psychometric properties and distinguished painful and non-painful states	Lints-Martindale et al.	2012
	Not allowing people near	Strong, +	Association of a global PASLAC-II score with other pain assessments (PASLAC, NOPPAIN, CNPI, PADE, PAINAD)	Chan et al.	2014
		Strong, +	Association of the PACSLAC scale and global pain intensity rating during pain events	Fuchs-Lacelle & Hadjistavropoulos	2004
	Not wanting to be touched	Strong, +	Association of the PACSLAC scale and global pain intensity rating during pain events	Fuchs-Lacelle & Hadjistavropoulos	2004
		Strong, +	Association of a global PASLAC-II score with other pain assessments (PASLAC, NOPPAIN, CNPI, PADE, PAINAD)	Chan et al.	2014
		Strong, +	Association of the CNPI and PATCIE scales in relation to movement	Richey et al.	2020
	Requesting attention	Weak/medium, +	Decrease in symptom due to intervention led to a decrease in pain.	Husebo, Ballard et al.	2013
	Social life	Weak/medium, +	Association of the Doloplus-2 with self-reported pain (VAS)	Pautex et al.	2007
		Weak/medium, +	Association of the Doloplus-2 with self-reported pain (VAS)	Pautex et al.	2007
		Not specified, +	More patients evaluated as experiencing pain with Doloplus-2 than with a single question	Torvik et al.	2010
	Striking out	Strong, +	Association of the CNPI and PATCIE scales in relation to movement	Richey et al.	2020
	Unsocial behavior	Strong, +	Association of the ePAT and APS scales	Atee et al.	2017
	Withdrawn	Strong, +	Association of the CNPI and PATCIE scales in relation to movement	Richey et al.	2020

Notes. APS= Abbey Pain Scale; BPSD= Behavioral and Psychological Symptoms of Dementia; CNPI= Checklist of Nonverbal Pain Indicators; ePAT= electronic Pain Assessment Tool; NOPPAIN= Non-Communicative Patient's Pain Assessment Instrument; PADE= Pain Assessment in Advanced Dementia; PAINAD= Pain Assessment in Advanced Dementia; PACSLAC= Pain Assessment Checklist for Seniors with Limited Ability to Communicate; PASLAC= Pain Assessment for Seniors with Limited Ability to Communicate; PASLAC-II= Pain Assessment for Seniors with Limited Ability to Communicate-II; PATCIE= Pain Assessment Tool for Cognitively Impaired Elderly; VAS= Visual Analog Scale.
Relevance and association direction of pain cue: + = positive, - = negative; Strong = large effect size, Weak/medium = small to medium effect size (Ellis, 2010), Not specified = study reported only on the significance and direction of the association, without statistical coefficients.

Table S2
Summary of relevant cues of pain in the "body movement/expressions" subcategory.

**Pain cue**			**Relevance of the cue**		**Study**	
**Category 1**	**Category 2**	**Category 3**	**Relevance and association direction of pain cue**	**Description of association**	**Authors**	**Year**
Body movement/ expressions	Body language	[General]	Weak/medium, +	Rated by inter-rater agreement as a "Good indication to judge pain"	van Iersel et al.	2006
			Strong, +	Association of total PAINAD and VAS pain scale	Warden et al.	2003
			Not specified, +	More behavioral pain observations during aversive than pleasant activity for pain and agitation groups in relation to controls	Mahoney & Peters	2008
		Change in body language	Weak/medium, +	Rated by inter-rater agreement as a "Good indication to judge pain"	van Iersel et al.	2006
			Weak/medium, +	Association between Abbey pain scale and self-report	Takai et. al.,	2014
	Body movement	[General]	Strong, +	Higher PACSLAC score during pain events. Other tools investigated all had adequate psychometric properties and distinguished painful and non-painful states	Lints-Martindale et al.	2012
			Strong, +	Association of the ePAT and APS scales	Atee et al.	2018
		Bracing	Weak/medium, +	Association between the CNPI and VDS scales during movement	Feldt	2000
			Strong, +	Pain intensity significantly predicted the pain behaviours sum score, but no significant effect in the dementia group alone.	Horgas et al.	2009
			Weak/medium, +	Pain intensity was predictive of the behaviour	Horgas et al.	2009
			Weak/medium, +	Marker importance as rated by the nursing staff	Cohen-Mansfield & Creedon	2002
		Difficulty chewing	Strong, +	Marker importance as rated by the nursing staff	Cohen-Mansfield & Creedon	2002
		Fidgeting	Strong, +	Association of the PACSLAC scale and global pain intensity rating during pain events	Fuchs-Lacelle & Hadjistavropoulos	2004
		Flinching and/or pulling away	Strong, +	Association of the PACSLAC scale and global pain intensity rating during pain events	Fuchs-Lacelle & Hadjistavropoulos	2004
			Strong, +	Association of a global PASLAC-II score with other pain assessments (PASLAC, NOPPAIN, CNPI, PADE, PAINAD)	Chan et al.	2014
		Freezing	Weak/medium, +	Marker present when pain was more likely, as rated by sufficient interrater agreement or intraclass correlation coefficient	Kunz et al.	2019
			Strong, +	Association of the ePAT and APS scales	Atee et al.	2017
		Gait changes	Strong, +	Association of the CNPI and PATCIE scales in relation to movement	Richey et al.	2020
		Hand-wringing	Weak/medium, +	Marker presence associated with presence of pain	Cervo et al.	2007
		Leg/arm movement	Strong, +	Association of the ePAT and APS scales	Atee et al.	2017
		Limping	Strong, +	Association of the PACSLAC scale and global pain intensity rating during pain events	Fuchs-Lacelle & Hadjistavropoulos	2004
			Strong, +	Association of a global PASLAC-II score with other pain assessments (PASLAC, NOPPAIN, CNPI, PADE, PAINAD)	Chan et al.	2014

	Weak/medium, +	Response frequency to "What are the signs or indicators of pain in noncommunicative elderly people with dementia?"	Cohen-Mansfield & Creedon	2002
	Strong, +	Marker importance as rated by the nursing staff	Cohen-Mansfield & Creedon	2002
Pacing	Weak/medium, +	Decrease in symptom due to intervention led to a decrease in pain.	Husebo, Ballard et al.	2013
	Strong, +	Association of the PACSLAC scale and global pain intensity rating during pain events	Fuchs-Lacelle & Hadjistavropoulos	2004
Pulling/moving away	Strong, +	Association of the ePAT and APS scales	Atee et al.	2017
	Strong, +	Association of the PACSLAC scale and global pain intensity rating during pain events	Fuchs-Lacelle & Hadjistavropoulos	2004
Reluctance to move	Strong, +	Association of the PACSLAC scale and global pain intensity rating during pain events	Fuchs-Lacelle & Hadjistavropoulos	2004
	Strong, +	Association of a global PASLAC-II score with other pain assessments (PASLAC, NOPPAIN, CNPI, PADE, PAINAD)	Chan et al.	2014
	Strong, +	Association of the CNPI and PATCIE scales in relation to movement	Richey et al.	2020
	Weak/medium, +	Response frequency to "What are the signs or indicators of pain in noncommunicative elderly people with dementia?"	Cohen-Mansfield & Creedon	2002
	Weak/medium, +	Marker importance as rated by the nursing staff	Cohen-Mansfield & Creedon	2002
Repetitive movements	Weak/medium, +	Marker importance as rated by the nursing staff	Cohen-Mansfield & Creedon	2002
	Strong, +	Marker rated as important in a recursive model	Burfield et al.	2012
	Strong, +	Marker importance as rated by the nursing staff	Cohen-Mansfield & Creedon	2002
Restlessness	Strong, +	Decrease in symptom due to intervention led to a decrease in pain.	Husebo, Ballard et al.	2013
	Strong, +	Association of the CNPI and PATCIE scales in relation to movement	Richey et al.	2020
	Strong, +	Association of the PACSLAC scale and global pain intensity rating during pain events	Fuchs-Lacelle & Hadjistavropoulos	2004
	Strong, +	Marker present when pain was more likely, as rated by sufficient interrater agreement or intraclass correlation coefficient	Kunz et al.	2019
	Weak/medium, +	Significant relationship between the degree of pain and the frequency of BPSD	Hodgson et al.	2014
	Weak/medium, +	Marker importance as rated by the nursing staff	Cohen-Mansfield & Creedon	2002
	Strong, +	Association of the ePAT and APS scales	Atee et al.	2017
Restlessness at rest	Strong, +	Association of the item on OPS-NVI and self-reported orofacial pain	van de Rijt et al.	2019
Restlessness while chewing	Weak/medium, +	Association of the item on OPS-NVI and self-reported orofacial pain	van de Rijt et al.	2019

Rigid movement	Weak/medium, +	Marker importance as rated by the nursing staff	Cohen-Mansfield & Creedon	2002
Rigid body	Weak/medium, +	Marker importance as rated by the nursing staff	Cohen-Mansfield & Creedon	2002
Rigid muscles	Weak/medium, +	Marker importance as rated by the nursing staff	Cohen-Mansfield & Creedon	2002
Rigidity	Weak/medium, +	Marker presence associated with presence of pain	Cervo et al.	2007
	Strong, +	Pain intensity significantly predicted the pain behaviours sum score, but no significant effect in the dementia group alone.	Horgas et al.	2009
	Weak/medium, +	Pain intensity was predictive of the behaviour	Horgas et al.	2009
	Strong, +	Association of a global PASLAC-II score with other pain assessments (PASLAC, NOPPAIN, CNPI, PADE, PAINAD)	Chan et al.	2014
	Strong, +	Association of the PACSLAC scale and global pain intensity rating during pain events	Fuchs-Lacelle & Hadjistavropoulos	2004
Rocking motion	Strong, +	Association of the CNPI and PATCIE scales in relation to movement	Richey et al.	2020
Rocking motion - head	Weak/medium, +	Response frequency to "What are the signs or indicators of pain in noncommunicative elderly people with dementia?"	Cohen-Mansfield & Creedon	2002
	Weak/medium, +	Marker importance as rated by the nursing staff	Cohen-Mansfield & Creedon	2002
Rubbing	Mixed results, +	Association between the CNPI and VDS scales during movement	Feldt	2000
	Mixed results, +	no sig. relation in comparing pain-free and CLBP participants; sig. more present in cognitively impaired participants that in cognitively intact	Shega et al.	2008
	Strong, +	Marker present when pain was more likely, as rated by sufficient interrater agreement or intraclass correlation coefficient	Kunz et al.	2019
	Strong, +	Association of the CNPI and PATCIE scales in relation to movement	Richey et al.	2020
	Strong, +	Pain intensity significantly predicted the pain behaviours sum score, but no significant effect in the dementia group alone.	Horgas et al.	2009
	Strong, +	Association of a global PASLAC-II score with other pain assessments (PASLAC, NOPPAIN, CNPI, PADE, PAINAD)	Chan et al.	2014
	Weak/medium, +	Pain intensity was predictive of the behaviour	Horgas et al.	2009
	Weak/medium, +	Association of the item on OPS-NVI and self-reported orofacial pain	van de Rijt et al.	2019
	Weak/medium, +	Association of the item on OPS-NVI and self-reported orofacial pain	van de Rijt et al.	2019
Shaking/Trembling	Strong, +	Association of a global PASLAC-II score with other pain assessments (PASLAC, NOPPAIN, CNPI, PADE, PAINAD)	Chan et al.	2014
	Strong, +	Association of the PACSLAC scale and global pain intensity rating during pain events	Fuchs-Lacelle & Hadjistavropoulos	2004
Shifting	Strong, +	Pain intensity significantly predicted the pain behaviours sum score, but no significant effect in the dementia group alone.	Horgas et al.	2009
	Weak/medium, +	Pain intensity was predictive of the behaviour	Horgas et al.	2009
Slow movement	Strong, +	Association of the PACSLAC scale and global pain intensity rating during pain events	Fuchs-Lacelle & Hadjistavropoulos	2004

		Strong, +	Association of a global PASLAC-II score with other pain assessments (PASLAC, NOPPAIN, CNPI, PADE, PAINAD)	Chan et al.	2014
	Tense body	Weak/medium, +	Marker presence associated with presence of pain	Cervo et al.	2007
		Strong, +	Association of the CNPI and PATCIE scales in relation to movement	Richey et al.	2020
	Thrashing	Strong, +	Association of the PACSLAC scale and global pain intensity rating during pain events	Fuchs-Lacelle & Hadjistavropoulos	2004
		Strong, +	Association of a global PASLAC-II score with other pain assessments (PASLAC, NOPPAIN, CNPI, PADE, PAINAD)	Chan et al.	2014
	Tossing and/or turning	Weak/medium, +	Response frequency to "What are the signs or indicators of pain in noncommunicative elderly people with dementia?"	Cohen-Mansfield & Creedon	2002
		Weak/medium, +	Marker importance as rated by the nursing staff	Cohen-Mansfield & Creedon	2002
		Mixed results, +	Association of total pain score to CMAI and NPI-NH	Husebo, Ostelo et al.	2014
	Touching a body part/area	Strong, +	Response frequency to "What are the signs or indicators of pain in noncommunicative elderly people with dementia?"	Cohen-Mansfield & Creedon	2002
		Strong, +	Association of the PACSLAC scale and global pain intensity rating during pain events	Fuchs-Lacelle & Hadjistavropoulos	2004
		Strong, +	Association of the CNPI and PATCIE scales in relation to movement	Richey et al.	2020
	Trembling	Weak/medium, +	Marker importance as rated by the nursing staff	Cohen-Mansfield & Creedon	2002
	Wincing	Weak/medium, +	Response frequency to "What are the signs or indicators of pain in noncommunicative elderly people with dementia?"	Cohen-Mansfield & Creedon	2002
		Strong, +	Marker importance as rated by the nursing staff	Cohen-Mansfield & Creedon	2002
Body parts and markers	[General]	Weak/medium, +	Marker rated as important in a recursive model	Burfield et al.	2012
		Strong, +	Association of the ePAT and APS scales	Atee et al.	2018
	Abdomen	Mixed results, +	Association of total pain score to CMAI and NPI-NH	Husebo, Ostelo et al.	2014
		Mixed results, +	MOBID-2 specific marker reduced after pain intervention	Sandvik et al.	2014
	Arms	Mixed results, +	Association of total pain score to CMAI and NPI-NH	Husebo, Ostelo et al.	2014
		Strong, +	MOBID-2 specific marker reduced after pain intervention	Sandvik et al.	2014
	Hands	Mixed results, +	Association of total pain score to CMAI and NPI-NH	Husebo, Ostelo et al.	2014
		Weak/medium, +	MOBID-2 specific marker reduced after pain intervention	Sandvik et al.	2014
	Head, mouth, neck	Mixed results, +	Association of total pain score to CMAI and NPI-NH	Husebo, Ostelo et al.	2014
		Weak/medium, +	MOBID-2 specific marker reduced after pain intervention	Sandvik et al.	2014
		Weak/medium, +	MOBID-2 specific marker reduced after pain intervention	Sandvik et al.	2014

	Heart, lung, chest wall	Mixed results, +	Association of total pain score to CMAI and NPI-NH	Husebo, Ostelo et al.	2014
	Legs	Mixed results, +	Association of total pain score to CMAI and NPI-NH	Husebo, Ostelo et al.	2014
		Mixed results, +	MOBID-2 specific marker reduced after pain intervention	Sandvik et al.	2014
	Pelvis, genital organs	Mixed results, +	Association of total pain score to CMAI and NPI-NH	Husebo, Ostelo et al.	2014
		Strong, +	MOBID-2 specific marker reduced after pain intervention	Sandvik et al.	2014
	Sitting	Strong, +	MOBID-2 specific marker reduced after pain intervention	Sandvik et al.	2014
	Skin	Mixed results, +	Association of total pain score to CMAI and NPI-NH	Husebo, Ostelo et al.	2014
		Mixed results, +	MOBID-2 specific marker reduced after pain intervention	Sandvik et al.	2014
	Turn over	Weak/medium, +	MOBID-2 specific marker reduced after pain intervention	Sandvik et al.	2014
Body positions / postures	Abnormal or awkward sitting / standing / walking	Strong, +	Association of the ePAT and APS scales	Atee et al.	2017
		Weak/medium, +	Response frequency to "What are the signs or indicators of pain in noncommunicative elderly people with dementia?"	Cohen-Mansfield & Creedon	2002
		Strong, +	Marker importance as rated by the nursing staff	Cohen-Mansfield & Creedon	2002
	Clenched fist	Strong, +	Association of the PACSLAC scale and global pain intensity rating during pain events	Fuchs-Lacelle & Hadjistavropoulos	2004
		Strong, +	Association of a global PASLAC-II score with other pain assessments (PASLAC, NOPPAIN, CNPI, PADE, PAINAD)	Chan et al.	2014
	Foetal position	Strong, +	Association of the PACSLAC scale and global pain intensity rating during pain events	Fuchs-Lacelle & Hadjistavropoulos	2004
		Strong, +	Association of a global PASLAC-II score with other pain assessments (PASLAC, NOPPAIN, CNPI, PADE, PAINAD)	Chan et al.	2014
	Guarding	Weak/medium, +	Marker sig. more present in patients with CLBP than in pain-free participants as well as in cognitively impaired participants than in cognitively intact	Shega et al.	2008
		Strong, +	Marker present when pain was more likely, as rated by sufficient interrater agreement or intraclass correlation coefficient	Kunz et al.	2019
		Strong, +	Association of the CNPI and PATCIE scales in relation to movement	Richey et al.	2020
		Strong, +	Pain intensity significantly predicted the pain behaviours sum score, but no significant effect in the dementia group alone.	Horgas et al.	2009
		Weak/medium, +	Pain intensity was predictive of the behaviour	Horgas et al.	2009
		Strong, +	Association of the PACSLAC scale and global pain intensity rating during pain events	Fuchs-Lacelle & Hadjistavropoulos	2004
		Strong, +	Association of a global PASLAC-II score with other pain assessments (PASLAC, NOPPAIN, CNPI, PADE, PAINAD)	Chan et al.	2014
		Strong, +	Association of the ePAT and APS scales	Atee et al.	2017
		Weak/medium, +	Association of the Doloplus-2 with self-reported pain (VAS)	Pautex et al.	2007
		Weak/medium, +	Association of the shortened Doloplus-2 with self-reported pain (VAS)	Pautex et al.	2007

		Not specified, +	More patients evaluated as experiencing pain with Doloplus-2 than with a single question	Torvik et al.	2010
	Poor posture	Weak/medium, +	Marker importance as rated by the nursing staff	Cohen-Mansfield & Creedon	2002
	Protective posture at rest	Weak/medium, +	Association of the Doloplus-2 with self-reported pain (VAS)	Pautex et al.	2007
		Weak/medium, +	Association of the shortened Doloplus-2 with self-reported pain (VAS)	Pautex et al.	2007
		Not specified, +	More patients evaluated as experiencing pain with Doloplus-2 than with a single question	Torvik et al.	2010
	Sitting	Mixed results, +	Association of total pain score to CMAI and NPI-NH	Husebo, Ostelo et al.	2014
Physical cues	Abnormal skin colour	Strong, +	Marker importance as rated by the nursing staff	Cohen-Mansfield & Creedon	2002
	Blood stains	Weak/medium, +	Response frequency to "What are the signs or indicators of pain in noncommunicative elderly people with dementia?"	Cohen-Mansfield & Creedon	2002
		Weak/medium, +	Marker importance as rated by the nursing staff	Cohen-Mansfield & Creedon	2002
	Heat from specific body part	Strong, +	Marker importance as rated by the nursing staff	Cohen-Mansfield & Creedon	2002
	Swollen joints	Strong, +	Marker importance as rated by the nursing staff	Cohen-Mansfield & Creedon	2002
	Tight belly	Strong, +	Marker importance as rated by the nursing staff	Cohen-Mansfield & Creedon	2002

Notes. APS = Abbey Pain Scale; BPSD = Behavioral and Psychological Symptoms of Dementia; CNPI = Checklist of Nonverbal Pain Indicators; CMAI = Cohen-Mansfield Agitation Inventory; Doloplus-2 = Doloplus-2 Pain Assessment Scale; ePAT = electronic Pain Assessment Tool; MOBID-2 = Mobilization-Observation-Behavior-Intensity-Dementia Pain Scale-2; NOPPAIN = Non-Communicative Patient's Pain Assessment Instrument; NPI-NH = Neuropsychiatric Inventory–Nursing Home Version; OPS-NVI = Orofacial Pain Scale–Nonverbal Individuals; PADE = Pain Assessment in Advanced Dementia; PAINAD = Pain Assessment in Advanced Dementia; PACSLAC = Pain Assessment Checklist for Seniors with Limited Ability to Communicate; PASLAC = Pain Assessment for Seniors with Limited Ability to Communicate; PASLAC-II = Pain Assessment for Seniors with Limited Ability to Communicate-II; PATCIE = Pain Assessment Tool for Cognitively Impaired Elderly; VAS = Visual Analog Scale; VDS = Verbal Descriptor Scale.
Relevance and association direction of pain cue: + = positive, - = negative; Strong = large effect size, Weak/medium = small to medium effect size (Ellis, 2010), Mixed results = study reported inconsistent findings, Not specified = study reported only on the significance and direction of the association, without statistical coefficients.

Table S3
Summary of relevant cues of pain in the "facial expressions" subcategory.

Pain cue			Relevance of the cue		Study	
**Category 1**	**Category 2**	**Category 3**	**Relevance and association direction of pain cue**	**Description of association**	**Authors**	**Year**
Facial expressions	Brows	Brow lowering	Strong, +	Patient sig. more likely to experience pain when marker was present	Atee et.al.	2022
			Strong, +	Markers were associated with observational pain scores in residents with dementia	Pu et al.	2024
			Strong, +	Association of the ePAT and APS scales	Atee et al.	2017
		Frowning	Weak/medium, +	Marker present when pain was more likely, as rated by sufficient interrater agreement or intraclass correlation coefficient	Kunz et al.	2019
			Strong, +	Association of the CNPI and PATCIE scales in relation to movement	Richey et al.	2020
			Strong, +	Association of the PACSLAC scale and global pain intensity rating during pain events	Fuchs-Lacelle & Hadjistavropoulos	2004
			Strong, +	Association of the item on OPS-NVI and self-reported orofacial pain	van de Rijt et al.	2019
			Strong, +	Association of the item on OPS-NVI and self-reported orofacial pain	van de Rijt et al.	2019
			Strong, +	Association of a global PASLAC-II score with other pain assessments (PASLAC, NOPPAIN, CNPI, PADE, PAINAD)	Chan et al.	2014
	Cheeks	Cheek raising	Strong, +	Association of the ePAT and APS scales	Atee et al.	2017
	Eyes	Changes in eyes	Strong, +	Association of the PACSLAC scale and global pain intensity rating during pain events	Fuchs-Lacelle & Hadjistavropoulos	2004
		Closing eyes	Strong, +	Patient sig. more likely to experience pain when marker was present	Atee et al.	2022
			Strong, +	Markers were associated with observational pain scores in residents with dementia	Pu et al.	2024
			Strong, +	Association of the ePAT and APS scales	Atee et al.	2017
			Strong, +	Association of a global PASLAC-II score with other pain assessments (PASLAC, NOPPAIN, CNPI, PADE, PAINAD)	Chan et al.	2014
		Dirty look	Strong, +	Association of the PACSLAC scale and global pain intensity rating during pain events	Fuchs-Lacelle & Hadjistavropoulos	2004
		Increased eye movement	Strong, +	Association of a global PASLAC-II score with other pain assessments (PASLAC, NOPPAIN, CNPI, PADE, PAINAD)	Chan et al.	2014
		Narrowing and/or closing eyes	Weak/medium, +	Marker present when pain was more likely, as rated by sufficient interrater agreement or intraclass correlation coefficient	Kunz et al.	2019
			Weak/medium, +	Association of the item on OPS-NVI and self-reported orofacial pain	van de Rijt et al.	2019
			Strong, +	Association of the item on OPS-NVI and self-reported orofacial pain	van de Rijt et al.	2019
		Teary eyes	Strong, +	Association of the PACSLAC scale and global pain intensity rating during pain events	Fuchs-Lacelle & Hadjistavropoulos	2004
		Tightening of eyelids	Strong, +	Patient sig. more likely to experience pain when marker was present	Atee et.al.	2022
			Strong, +	Association of the ePAT and APS scales	Atee et al.	2017

Forehead	Creasing forehead	Strong, +	Association of a global PASLAC-II score with other pain assessments (PASLAC, NOPPAIN, CNPI, PADE, PAINAD)	Chan et al.	2014
		Strong, +	Association of the PACSLAC scale and global pain intensity rating during pain events	Fuchs-Lacelle & Hadjistavropoulos	2004
Jaw	Restricting jaw movement while chewing	Strong, +	Association of the item on OPS-NVI and self-reported orofacial pain	van de Rijt et al.	2019
Lips, mouth	Clenching teeth	Strong, +	Association of the PACSLAC scale and global pain intensity rating during pain events	Fuchs-Lacelle & Hadjistavropoulos	2004
		Strong, +	Association of the CNPI and PATCIE scales in relation to movement	Richey et al.	2020
	Drooling	Strong, +	Association of the item on OPS-NVI and self-reported orofacial pain	van de Rijt et al.	2019
	Horizontal mouth stretch	Strong, +	Patient sig. more likely to experience pain when marker was present	Atee et al.	2022
		Weak/medium, +	Markers were associated with observational pain scores in residents with dementia	Pu et al.	2024
		Strong, +	Association of the ePAT and APS scales	Atee et al.	2017
	Opening mouth	Strong, +	Association of a global PASLAC-II score with other pain assessments (PASLAC, NOPPAIN, CNPI, PADE, PAINAD)	Chan et al.	2014
		Weak/medium, +	Association of the item on OPS-NVI and self-reported orofacial pain	van de Rijt et al.	2019
		Weak/medium, +	Marker present when pain was more likely, as rated by sufficient interrater agreement or intraclass correlation coefficient	Kunz et al.	2019
		Strong, +	Association of the PACSLAC scale and global pain intensity rating during pain events	Fuchs-Lacelle & Hadjistavropoulos	2004
	Parting lips	Weak/medium, +	Patient sig. more likely to experience pain when marker was present	Atee et al.	2022
		Strong, +	Markers were associated with observational pain scores in residents with dementia	Pu et al.	2024
		Strong, +	Association of the ePAT and APS scales	Atee et al.	2017
	Pulling at corner lip	Strong, +	Association of the ePAT and APS scales	Atee et al.	2017
		Weak/medium, +	Patient sig. more likely to experience pain when marker was present	Atee et al.	2022
	Raising of upper lip	Strong, +	Markers were associated with observational pain scores in residents with dementia	Pu et al.	2024
		Strong, +	Association of the ePAT and APS scales	Atee et al.	2017
		Strong, +	Patient sig. more likely to experience pain when marker was present	Atee et al.	2022
		Strong, +	Marker present when pain was more likely, as rated by sufficient interrater agreement or intraclass correlation coefficient	Kunz et al.	2019
Nose	Screwing up nose	Strong, +	Association of the PACSLAC scale and global pain intensity rating during pain events	Fuchs-Lacelle & Hadjistavropoulos	2004
	Wrinkling nose	Weak/medium, +	Patient sig. more likely to experience pain when marker was present	Atee et al.	2022
		Strong, +	Association of the ePAT and APS scales	Atee et al.	2017
		Strong, +	Markers were associated with observational pain scores in residents with dementia	Pu et al.	2024

Whole face	[General]	Strong, +	Action units for the whole face showed higher activation when pain was present	Atee et al.	2022
		Strong, +	Association of the ePAT and APS scales	Atee et al.	2018
	Change in colour	Strong, +	Marker importance as rated by the nursing staff	Cohen-Mansfield & Creedon	2002
	Facial expression	Not specified, +	More patients evaluated as experiencing pain with Doloplus-2 than with a single question	Torvik et al.	2010
		Strong, +	Association between Abbey pain scale and self-report	Takai et al.	2014
		Not specified, +	More behavioral pain observations during aversive than pleasant activity for pain and agitation groups in relation to controls	Mahoney & Peters	2008
		Strong, +	Rated by inter-rater agreement as a "Good indication to judge pain"	van Iersel et al.	2006
		Strong, +	Higher PACSLAC score during pain events. Other tools investigated all had adequate psychometric properties and distinguished painful and non-painful states	Lints-Martindale et al.	2012
		Strong, +	Association of total PAINAD and VAS pain scale	Warden et al.	2003
	Facial expression - specific	Strong, +	Rated by inter-rater agreement as a "Good indication to judge pain"	van Iersel et al.	2006
		Strong, +	Association of a global PASLAC-II score with other pain assessments (PASLAC, NOPPAIN, CNPI, PADE, PAINAD)	Chan et al.	2014
	Fearful expression	Weak/medium, +	Marker presence associated with presence of pain	Cervo et al.	2007
	Flushed, red face	Strong, +	Association of the PACSLAC scale and global pain intensity rating during pain events	Fuchs-Lacelle & Hadjistavropoulos	2004
	Gloomy facial expression	Strong, +	Association of the CNPI and PATCIE scales in relation to movement	Richey et al.	2020
	Grim face	Strong, +	Association of the PACSLAC scale and global pain intensity rating during pain events	Fuchs-Lacelle & Hadjistavropoulos	2004
	Grimacing	Weak/medium, +	Response frequency to "What are the signs or indicators of pain in noncommunicative elderly people with dementia?"	Cohen-Mansfield & Creedon	2002
		Weak/medium, +	Marker importance as rated by the nursing staff	Cohen-Mansfield & Creedon	2002
		Mixed results, +	Association between the CNPI and VDS scales during movement	Feldt	2000
		Mixed results, +	Marker sig. more present in patients with CLBP than in pain-free participants as well as in cognitively impaired participants than in cognitively intact	Shega et al.	2008
		Strong, +	Association of the CNPI and PATCIE scales in relation to movement	Richey et al.	2020
		Strong, +	Association of a global PASLAC-II score with other pain assessments (PASLAC, NOPPAIN, CNPI, PADE, PAINAD)	Chan et al.	2014
		Strong, +	Association of the PACSLAC scale and global pain intensity rating during pain events	Fuchs-Lacelle & Hadjistavropoulos	2004
		Strong, +	Pain intensity significantly predicted the pain behaviours sum score, but no significant effect in the dementia group alone.	Horgas et al.	2009

	Strong, +	Pain intensity was predictive of the behaviour	Horgas et al.	2009
Looking tense	Weak/medium, +	Marker present when pain was more likely, as rated by sufficient interrater agreement or intraclass correlation coefficient	Kunz et al.	2019
Pain expression	Weak/medium, +	Association of the Doloplus-2 with self-reported pain (VAS)	Pautex et al.	2007
	Strong, +	Association of a global PASLAC-II score with other pain assessments (PASLAC, NOPPAIN, CNPI, PADE, PAINAD)	Chan et al.	2014
	Strong, +	Association of the PACSLAC scale and global pain intensity rating during pain events	Fuchs-Lacelle & Hadjistavropoulos	2004
Pale face	Strong, +	Association of the PACSLAC scale and global pain intensity rating during pain events	Fuchs-Lacelle & Hadjistavropoulos	2004
Pale or flushed/red face	Strong, +	Association of the ePAT and APS scales	Atee et al.	2017
Prkachin & Solomon Pain Index	Weak/medium, +	Better than baseline pain estimation model on faces of people with dementia	Rezaei et al.	2021
Sad expression/look	Strong, +	Marker rated as important in a recursive model	Burfield et al.	2012
	Mixed results, +	Association of the PACSLAC scale and global pain intensity rating during pain events	Fuchs-Lacelle & Hadjistavropoulos	2004
Scared expression	Weak/medium, +	Marker presence associated with presence of pain	Cervo et al.	2007
Tighter face	Strong, +	Association of a global PASLAC-II score with other pain assessments (PASLAC, NOPPAIN, CNPI, PADE, PAINAD)	Chan et al.	2014
	Strong, +	Association of the PACSLAC scale and global pain intensity rating during pain events	Fuchs-Lacelle & Hadjistavropoulos	2004
Wincing	Strong, +	Association of a global PASLAC-II score with other pain assessments (PASLAC, NOPPAIN, CNPI, PADE, PAINAD)	Chan et al.	2014
	Strong, +	Association of the PACSLAC scale and global pain intensity rating during pain events	Fuchs-Lacelle & Hadjistavropoulos	2004

Notes. APS = Abbey Pain Scale, BPSD = Behavioral and Psychological Symptoms of Dementia, CMAI = Cohen-Mansfield Agitation Inventory, CNPI = Checklist of Nonverbal Pain Indicators, CLBP = Chronic Low Back Pain, Doloplus-2 = Doloplus-2 Pain Assessment Scale, ePAT = electronic Pain Assessment Tool, MOBID-2 = Mobilization-Observation-Behavior-Intensity-Dementia Pain Scale-2, NOPPAIN = Nursing Home Pain Scale, NPI-NH = Neuropsychiatric Inventory–Nursing Home version, OPS-NVI = Orofacial Pain Scale–Nonverbal Individuals, PADE = Pain Assessment for Dementing Elderly, PAINAD = Pain Assessment in Advanced Dementia Scale, PACSLAC = Pain Assessment Checklist for Seniors with Limited Ability to Communicate, PASLAC-II = Pain Assessment for Seniors with Limited Ability to Communicate II, PATCIE = Pain Assessment Tool for Cognitive Impairment and Elderly, VAS = Visual Analog Scale, VDS = Verbal Descriptor Scale.

Relevance and association direction of pain cue: + = positive, - = negative; Strong = large effect size, Weak/medium = small to medium effect size (Ellis, 2010), Mixed results = study reported inconsistent findings, Not specified = study reported only on the significance and direction of the association, without statistical coefficients.

Table S4

Summary of relevant cues of pain in the "medical status - somatic" subcategory.

**Pain cue**			**Relevance of the cue**		**Study**	
**Category 1**	**Category 2**	**Category 3**	**Relevance and association direction of pain cue**	**Description of association**	**Authors**	**Year**
Medical status - somatic	Injuries	Dislocated limbs	Strong, +	Marker importance as rated by the nursing staff	Cohen-Mansfield & Creedon	2002
		Injuries	Strong, +	Association of the ePAT and APS scales	Atee et al.	2017
		Physical changes	Weak/medium, +	Rated by inter-rater agreement as a "Good indication to judge pain"	van Iersel et al.	2006
	Medical conditions	One leg shorter	Weak/medium, +	Marker importance as rated by the nursing staff	Cohen-Mansfield & Creedon	2002
		Painful medical conditions	Strong, +	Association of the ePAT and APS scales	Atee et al.	2017
			Mixed results, +	No significant differences between pleasant and aversive activities in pain and agitation groups. During aversive activity, the combined group had a significantly higher score than the null group.	Mahoney & Peters	2008

Notes. APS = Abbey Pain Scale; ePAT= electronic Pain Assessment Tool.

Relevance and association direction of pain cue: + = positive, - = negative; Strong = large effect size, Weak/medium = small to medium effect size (Ellis, 2010), Mixed results = study reported inconsistent findings.

Table S5
Summary of relevant cues of pain in the "mental state / mood" subcategory.

Pain cue			Relevance of the cue		Study	
**Category 1**	**Category 2**	**Category 3**	**Relevance and association direction of pain cue**	**Description of association**	**Authors**	**Year**
Mental state / mood	Mental state	Changes in mental status	Strong, +	Association of a global PASLAC-II score with other pain assessments (PASLAC, NOPPAIN, CNPI, PADE, PAINAD)	Chan et al.	2014
			Strong, +	Higher PACSLAC score during pain events. Other tools investigated all had adequate psychometric properties and distinguished painful and non-painful states	Lints-Martindale et al.	2012
			Strong, +	Response frequency to "What are the signs or indicators of pain in noncommunicative elderly people with dementia?"	Cohen-Mansfield & Creedon	2002
		Confusion	Strong, +	Association of the ePAT and APS scales	Atee et al.	2017
			Strong, +	Association of the CNPI and PATCIE scales in relation to movement	Richey et al.	2020
			Strong, +	Association of the PACSLAC scale and global pain intensity rating during pain events	Fuchs-Lacelle & Hadjistavropoulos	2004
		Delusions	Weak/medium, +	Significant relationship between the degree of pain and the frequency of BPSD	Hodgson et al.	2014
		Distressed	Strong, +	Association of the ePAT and APS scales	Atee et al.	2017
	Mood indicators	Agitation	Weak/medium, +	Marker importance as rated by the nursing staff	Cohen-Mansfield & Creedon	2002
			Strong, +	Association of the PACSLAC scale and global pain intensity rating during pain events	Fuchs-Lacelle & Hadjistavropoulos	2004
			Weak/medium, +	Significant relationship between the degree of pain and the frequency of BPSD	Hodgson et al.	2014
		Anger	Strong, +	Association of the PACSLAC scale and global pain intensity rating during pain events	Fuchs-Lacelle & Hadjistavropoulos	2004
		Anxiousness, nervousness	Weak/medium, +	Significant relationship between the degree of pain and the frequency of BPSD	Hodgson et al.	2014
			Strong, +	Association of the PACSLAC scale and global pain intensity rating during pain events	Fuchs-Lacelle & Hadjistavropoulos	2004
		Depression	Weak/medium, +	Marker importance as rated by the nursing staff	Cohen-Mansfield & Creedon	2002
			Weak/medium, +	Response frequency to "What are the signs or indicators of pain in noncommunicative elderly people with dementia?"	Cohen-Mansfield & Creedon	2002
		Fear	Strong, +	Association of the ePAT and APS scales	Atee et al.	2017
		Frustrated	Strong, +	Association of the PACSLAC scale and global pain intensity rating during pain events	Fuchs-Lacelle & Hadjistavropoulos	2004
		Irritable	Strong, +	Association of the PACSLAC scale and global pain intensity rating during pain events	Fuchs-Lacelle & Hadjistavropoulos	2004

		Strong, +	Marker importance as rated by the nursing staff	Cohen-Mansfield & Creedon	2002
		Strong, +	Association of the CNPI and PATCIE scales in relation to movement	Richey et al.	2020
	Mood changes	Weak/medium, +	Marker rated as important in a recursive model	Burfield et al.	2012
	Moodiness	Weak/medium, +	Marker importance as rated by the nursing staff	Cohen-Mansfield & Creedon	2002
		Strong, +	Association of the PACSLAC scale and global pain intensity rating during pain events	Fuchs-Lacelle & Hadjistavropoulos	2004

Notes. APS = Abbey Pain Scale, BPSD = Behavioral and Psychological Symptoms of Dementia, CMAI = Cohen-Mansfield Agitation Inventory, CNPI = Checklist of Nonverbal Pain Indicators, CLBP = Chronic Low Back Pain, Doloplus-2 = Doloplus-2 Pain Assessment Scale, ePAT = electronic Pain Assessment Tool, MOBID-2 = Mobilization-Observation-Behavior-Intensity-Dementia Pain Scale-2, NOPPAIN = Nursing Home Pain Scale, NPI-NH = Neuropsychiatric Inventory–Nursing Home version, OPS-NVI = Orofacial Pain Scale–Nonverbal Individuals, PADE = Pain Assessment for Dementing Elderly, PAINAD = Pain Assessment in Advanced Dementia Scale, PACSLAC = Pain Assessment Checklist for Seniors with Limited Ability to Communicate, PASLAC-II = Pain Assessment for Seniors with Limited Ability to Communicate II, PATCIE = Pain Assessment Tool for Cognitive Impairment and Elderly, VAS = Visual Analog Scale, VDS = Verbal Descriptor Scale.

Relevance and association direction of pain cue: + = positive, - = negative; Strong = large effect size, Weak/medium = small to medium effect size (Ellis, 2010).

Table S6

Summary of relevant cues of pain in the "physiology" subcategory.

Pain cue			Relevance of the cue		Study	
**Category 1**	**Category 2**	**Category 3**	**Relevance and association direction of pain cue**	**Description of association**	**Authors**	**Year**
Physiology	Body temperature	Cold / feverish	Strong, +	Association of the ePAT and APS scales	Atee et al.	2017
		Cold & clammy	Strong, +	Association of the PACSLAC scale and global pain intensity rating during pain events	Fuchs-Lacelle & Hadjistavropoulos	2004
		Sweating	Strong, +	Association of the ePAT and APS scales	Atee et al.	2017
			Strong, +	Marker importance as rated by the nursing staff	Cohen-Mansfield & Creedon	2002
			Strong, +	Association of the PACSLAC scale and global pain intensity rating during pain events	Fuchs-Lacelle & Hadjistavropoulos	2004
	Breathing	Ease of breathing	Not specified, -	More behavioral pain observations during aversive than pleasant activity for pain and agitation groups in relation to controls	Mahoney & Peters	2008
			Strong, -	Association of total PAINAD and VAS pain scale	Warden et al.	2003
		Gasping/ breathing loudly	Weak/medium, +	Marker importance as rated by the nursing staff	Cohen-Mansfield & Creedon	2002
			Strong, +	Association of a global PASLAC-II score with other pain assessments (PASLAC, NOPPAIN, CNPI, PADE, PAINAD)	Chan et al.	2014
		Rapid breathing	Strong, +	Association of the ePAT and APS scales	Atee et al.	2017
	Other	Physical change	Weak/medium, +	Higher score in pain vs no pain group, as well as "severe" MMSE group;	Takai et al.	2014
		Vomiting	Weak/medium, +	Marker importance as rated by the nursing staff	Cohen-Mansfield & Creedon	2002
	Vital signs - several	Changes in vital signs - general	Strong, +	Marker importance as rated by the nursing staff	Cohen-Mansfield & Creedon	2002
			Strong, +	Higher score in pain vs no pain group, as well as "very severe" MMSE group:	Takai et. al.,	2014
			Mixed results, +	More behavioral pain observations during aversive than pleasant activity for pain and agitation groups in relation to controls	Mahoney & Peters	2008

Notes. APS = Abbey Pain Scale, BPSD = Behavioral and Psychological Symptoms of Dementia, CNPI = Checklist of Nonverbal Pain Indicators, ePAT = electronic Pain Assessment Tool, MMSE = Mini-Mental State Examination, NOPPAIN = Nursing Home Pain Scale, PADE = Pain Assessment for Dementing Elderly, PAINAD = Pain Assessment in Advanced Dementia Scale, PACSLAC = Pain Assessment Checklist for Seniors with Limited Ability to Communicate, PASLAC-II = Pain Assessment for Seniors with Limited Ability to Communicate II, VAS = Visual Analog Scale. Relevance and association direction of pain cue: + = positive, - = negative; Strong = large effect size, Weak/medium = small to medium effect size (Ellis, 2010), Mixed results = study reported inconsistent findings, Not specified = study reported only on the significance and direction of the association, without statistical coefficients.

Table S7
Summary of relevant cues of pain in the "speech, language and sounds" subcategory.

Pain cue			Relevance of the cue		Study	
**Category 1**	**Category 2**	**Category 3**	**Relevance and association direction of pain cue**	**Description of association**	**Authors**	**Year**
Speech, language and sounds	[Other]	[General]	Strong, +	Association of the ePAT and APS scales	Atee et al.	2018
		Pain frequency	Weak/medium, +	Marker rated as important in a recursive model	Burfield et al.	2012
		Pain intensity	Weak/medium, +	Marker rated as important in a recursive model	Burfield et al.	2012
		Quietness	Weak/medium, +	Marker importance as rated by the nursing staff	Cohen-Mansfield & Creedon	2002
	Complaining	Somatic complaints	Weak/medium, +	Association of the Doloplus-2 with self-reported pain (VAS)	Pautex et al.	2007
			Weak/medium, +	Association of the shortened Doloplus-2 with self-reported pain (VAS)	Pautex et al.	2007
			Not specified, +	More patients evaluated as experiencing pain with Doloplus-2 than with a single question	Torvik et al.	2010
		Verbal complaints	Strong, +	Decrease in symptom due to intervention led to a decrease in pain.	Husebo, Ballard et al.	2013
			Strong, +	Marker present when pain was more likely, as rated by sufficient interrater agreement or intraclass correlation coefficient	Kunz et al.	2019
			Mixed results, +	Association between the CNPI and VDS scales during movement	Feldt	2000
			Strong, +	Pain intensity significantly predicted the pain behaviours sum score, but no significant effect in the dementia group alone.	Horgas et al.	2009
			Strong, +	Pain intensity was predictive of the behaviour	Horgas et al.	2009
	Inappropriate verbal behavior	Verbal aggression	Weak/medium, +	Decrease in symptom due to intervention led to a decrease in pain.	Husebo, Ballard et al.	2013
			Strong, +	Association of the PACSLAC scale and global pain intensity rating during pain events	Fuchs-Lacelle & Hadjistavropoulos	2004
		Verbally offensive	Strong, +	Association of the ePAT and APS scales	Atee et al.	2017
	Language	Calling out	Strong, +	Association of the PACSLAC scale and global pain intensity rating during pain events	Fuchs-Lacelle & Hadjistavropoulos	2004
			Weak/medium, +	Marker presence associated with presence of pain	Cervo et al.	2007
		Negative statements	Weak/medium, +	Marker rated as important in a recursive model	Burfield et al.	2012
		Negativism	Strong, +	Decrease in symptom due to intervention led to a decrease in pain.	Husebo, Ballard et al.	2013
		Repetitive verbalizations	Weak/medium, +	Decrease in symptom due to intervention led to a decrease in pain.	Husebo, Ballard et al.	2013
			Weak/medium, +	Marker rated as important in a recursive model	Burfield et al.	2012
			Strong, +	Larger effect size and predictability of pain scores when marker present	Hoti et al.	2023

	Requesting help repeatedly	Strong, +	Association of the ePAT and APS scales	Atee et al.	2017
Non-verbal expression	[General]	Weak/medium, +	Association between Abbey pain scale and self-report. Abbey scale distinguishes level of pain across MMSE subgroups	Takai et al.	2014
		Mixed results, +	Association between the CNPI and VDS scales during movement	Feldt	2000
		Strong, +	Higher PACSLAC score during pain events. Other tools investigated all had adequate psychometric properties and distinguished painful and non-painful states	Lints-Martindale et al.	2012
	Crying	Strong, +	Larger effect size and predictability of pain scores when marker present	Hoti et al.	2023
		Strong, +	Marker rated as important in a recursive model	Burfield et al.	2012
		Strong, +	Association of the CNPI and PATCIE scales in relation to movement	Richey et al.	2020
		Strong, +	Association of the ePAT and APS scales	Atee et al.	2017
		Strong, +	Association of the PACSLAC scale and global pain intensity rating during pain events	Fuchs-Lacelle & Hadjistavropoulos	2004
		Weak/medium, +	Marker importance as rated by the nursing staff	Cohen-Mansfield & Creedon	2002
		Strong, +	Association of a global PASLAC-II score with other pain assessments (PASLAC, NOPPAIN, CNPI, PADE, PAINAD)	Chan et al.	2014
	Groaning	Strong, +	Larger effect size and predictability of pain scores when marker present	Hoti et al.	2023
		Strong, +	Association of the ePAT and APS scales	Atee et al.	2017
		Strong, +	Marker present when pain was more likely, as rated by sufficient interrater agreement or intraclass correlation coefficient	Kunz et al.	2019
		Strong, +	Association of the CNPI and PATCIE scales in relation to movement	Richey et al.	2020
	Grunting	Strong, +	Association of the PACSLAC scale and global pain intensity rating during pain events	Fuchs-Lacelle & Hadjistavropoulos	2004
		Strong, +	Association of a global PASLAC-II score with other pain assessments (PASLAC, NOPPAIN, CNPI, PADE, PAINAD)	Chan et al.	2014
		Strong, +	Association of the CNPI and PATCIE scales in relation to movement	Richey et al.	2020
	Howling	Strong, +	Larger effect size and predictability of pain scores when marker present	Hoti et al.	2023
		Strong, +	Association of the ePAT and APS scales	Atee et al.	2017
	Moaning	Strong, +	Larger effect size and predictability of pain scores when marker present	Hoti et al.	2023
		Strong, +	Association of the CNPI and PATCIE scales in relation to movement	Richey et al.	2020
		Strong, +	Marker presence associated with presence of pain	Cervo et al.	2007
		Strong, +	Association of the ePAT and APS scales	Atee et al.	2017
	Moaning & groaning	Strong, +	Association of the PACSLAC scale and global pain intensity rating during pain events	Fuchs-Lacelle & Hadjistavropoulos	2004
		Strong, +	Association of a global PASLAC-II score with other pain assessments (PASLAC, NOPPAIN, CNPI, PADE, PAINAD)	Chan et al.	2014
	Mumbling	Strong, +	Association of the PACSLAC scale and global pain intensity rating during pain events	Fuchs-Lacelle & Hadjistavropoulos	2004

		Strong, +	Marker present when pain was more likely, as rated by sufficient interrater agreement or intraclass correlation coefficient	Kunz et al.	2019
	Negative vocalizations	Weak/medium, +	Response frequency to "What are the signs or indicators of pain in noncommunicative elderly people with dementia?"	Cohen-Mansfield & Creedon	2002
		Weak/medium, +	Marker importance as rated by the nursing staff	Cohen-Mansfield & Creedon	2002
		Weak/medium, +	Rated by inter-rater agreement as a "Good indication to judge pain"	van Iersel et al.	2006
		Strong, +	Association of total PAINAD and VAS pain scale	Warden et al.	2003
		Strong, +	Good psychometric properties of PACI for use by untrained professionals	Kaasalainen et al.	2011b
		Not specified, +	More behavioral pain observations during aversive than pleasant activity for pain and agitation groups in relation to controls	Mahoney & Peters	2008
		Weak/medium, +	Rated by inter-rater agreement as a "Good indication to judge pain"	van Iersel et al.	2006
	Screaming	Weak/medium, +	Response frequency to "What are the signs or indicators of pain in noncommunicative elderly people with dementia?"	Cohen-Mansfield & Creedon	2002
		Strong, +	Larger effect size and predictability of pain scores when marker present	Hoti et al.	2023
		Strong, +	Association of the ePAT and APS scales	Atee et al.	2017
		Weak/medium, +	Marker importance as rated by the nursing staff	Cohen-Mansfield & Creedon	2002
		Strong, +	Association of the PACSLAC scale and global pain intensity rating during pain events	Fuchs-Lacelle & Hadjistavropoulos	2004
	Shouting	Strong, +	Marker present when pain was more likely, as rated by sufficient interrater agreement or intraclass correlation coefficient	Kunz et al.	2019
	Sighing	Strong, +	Larger effect size and predictability of pain scores when marker present	Hoti et al.	2023
		Strong, +	Association of the CNPI and PATCIE scales in relation to movement	Richey et al.	2020
		Strong, +	Association of the ePAT and APS scales	Atee et al.	2017
		Strong, +	Pain intensity significantly predicted the pain behaviours sum score, but no significant effect in the dementia group alone.	Horgas et al.	2009
		Strong, +	Pain intensity was predictive of the behaviour	Horgas et al.	2009
	Whining	Strong, +	Association of the CNPI and PATCIE scales in relation to movement	Richey et al.	2020
	Yelling	Strong, +	Association of the CNPI and PATCIE scales in relation to movement	Richey et al.	2020
Speech	Loud talk	Strong, +	Larger effect size and predictability of pain scores when marker present	Hoti et al.	2023
		Strong, +	Association of the ePAT and APS scales	Atee et al.	2017
Verbal expression	Specific pain sounds/words	Strong, +	Association of a global PASLAC-II score with other pain assessments (PASLAC, NOPPAIN, CNPI, PADE, PAINAD)	Chan et al.	2014
		Strong, +	Association of the PACSLAC scale and global pain intensity rating during pain events	Fuchs-Lacelle & Hadjistavropoulos	2004
		Strong, +	Larger effect size and predictability of pain scores when marker present	Hoti et al.	2023
		Strong, +	Association of the ePAT and APS scales	Atee et al.	2017
		Strong, +	Marker present when pain was more likely, as rated by sufficient interrater agreement or intraclass correlation coefficient	Kunz et al.	2019

	Strong, +	Good psychometric properties of PACI for use by untrained professionals	Kaasalainen et al.	2011b

Notes. APS = Abbey Pain Scale, BPSD = Behavioral and Psychological Symptoms of Dementia, CNPI = Checklist of Nonverbal Pain Indicators, Doloplus-2 = Doloplus-2 Pain Assessment Scale, ePAT = electronic Pain Assessment Tool, MMSE = Mini-Mental State Examination, NOPPAIN = Nursing Home Pain Scale, PADE = Pain Assessment for Dementing Elderly, PAINAD = Pain Assessment in Advanced Dementia Scale, PACI = Pain Assessment in Cognitively Impaired, PACSLAC = Pain Assessment Checklist for Seniors with Limited Ability to Communicate, PASLAC-II = Pain Assessment for Seniors with Limited Ability to Communicate II, PATCIE = Pain Assessment Tool for Cognitive Impairment and Elderly, VAS = Visual Analog Scale, VDS = Verbal Descriptor Scale.

Relevance and association direction of pain cue: + = positive, - = negative; Strong = large effect size, Weak/medium = small to medium effect size (Ellis, 2010), Mixed results = study reported inconsistent findings, Not specified = study reported only on the significance and direction of the association, without statistical coefficients.

Table S8
Summary of irrelevant cues or cues with no reported statistical information across categories.

Pain cue			Relevance of the cue		Study	
**Category 1**	**Category 2**	**Category 3**	**Relevance of pain cue**	**Description of association**	**Authors**	**Year**
Behavior	Active behavior	Falls	Not significant	Response frequency to “What are the signs or indicators of pain in noncommunicative elderly people with dementia?”	Cohen-Mansfield & Creedon	2002
		Falls	Not significant	no relationship to pain	Husebo, Ballard et al.	2013
		Scratching	Not significant	no relationship to pain	Husebo, Ballard et al.	2013
	Behavioral change	[General]	No information	Symptom occurrence as associated with pain	Lundin et al.	2021
			No information	Symptom occurrence as associated with pain	Karlsson et al.	2015
			No information	Symptom occurrence as associated with pain	Jansen et al.	2017
			Not significant	Inter-rater agreement ("Good indication to judge pain")	van Iersel et al.	2006
		Changes in appetite	Not significant	Response frequency to “What are the signs or indicators of pain in noncommunicative elderly people with dementia?”	Cohen-Mansfield & Creedon	2002
		Changes in routines	Not significant	Behavioral pain observations during aversive than pleasant activity for pain and agitation groups in relation to controls	Mahoney & Peters	2008
		Decrease in activity	Not significant	Response frequency to “What are the signs or indicators of pain in noncommunicative elderly people with dementia?”	Cohen-Mansfield & Creedon	2002
		Stopping an activity	Not significant	No sig. effect for the dementia group	Horgas et al.	2009
	Inappropriate behavior	Biting	Not significant	No relationship to pain	Husebo, Ballard et al.	2013
		Eating inappropriate substances	Not significant	No relationship to pain	Husebo, Ballard et al.	2013
		Grabbing	Not significant	No relationship to pain	Husebo, Ballard et al.	2013
		Hiding	Not significant	No relationship to pain	Husebo, Ballard et al.	2013
		Hitting	Not significant	No relationship to pain	Husebo, Ballard et al.	2013
		Hoarding	Not significant	No relationship to pain	Husebo, Ballard et al.	2013
		Hurting self or others	Not significant	No relationship to pain	Husebo, Ballard et al.	2013
		Kicking	Not significant	No relationship to pain	Husebo, Ballard et al.	2013

		Pushing	Not significant	No relationship to pain	Husebo, Ballard et al.	2013
		Robing/disrobing	Not significant	No relationship to pain	Husebo, Ballard et al.	2013
		Spitting	Not significant	No relationship to pain	Husebo, Ballard et al.	2013
		Tearing things	Not significant	No relationship to pain	Husebo, Ballard et al.	2013
		Throwing things	Not significant	No relationship to pain	Husebo, Ballard et al.	2013
		Trying to leave/get to a different place	Not significant	No relationship to pain	Husebo, Ballard et al.	2013
	Mood-related behaviour	Pleasant behavior	Not significant	Association for the absence of pain	Cervo et al.	2007
	Social behavior	Consolability	Not significant	Inter-rater agreement ("Good indication to judge pain")	van Iersel et al.	2006
		Physical sexual advances	Not significant	No relationship to pain	Husebo, Ballard et al.	2013
Body movement/ expressions	Body language	[General]	No information	Reports of formal caregivers	Kaasalainen et al.	2011a
			No information	Symptom occurrence as associated with pain	Jansen et al.	2017
	Body movement	Bracing	Not significant	No sig. relation in comparing pain-free and CLBP participants;	Shega et al.	2008
		Decreased movement	No information	Symptom occurrence as associated with pain	Lundin et al.	2021
		Ease of movement	Not significant	Association for the absence of pain	Cervo et al.	2007
		Pacing	Not significant	Marker importance as rated by the nursing staff	Cohen-Mansfield & Creedon	2002
		Reluctance to move	No information	Symptom occurrence as associated with pain	Karlsson et al.	2015
		Repetitive movements	Not significant	No relationship to pain	Husebo, Ballard et al.	2013
		Rigidity	Not significant	Response frequency to “What are the signs or indicators of pain in noncommunicative elderly people with dementia?”	Cohen-Mansfield & Creedon	2002
		Rubbing	Not significant	Marker importance as rated by the nursing staff	Cohen-Mansfield & Creedon	2002
			No information	Validity of the marker in the PACI tool	Kaasalainen et al.	2011b
		Tense body	No information	Symptom occurrence as associated with pain	Lundin et al.	2021
		Trembling	Not significant	Response frequency to “What are the signs or indicators of pain in noncommunicative elderly people with dementia?”	Cohen-Mansfield & Creedon	2002

	Body positions/postures	Guarding	No information	Validity of the marker in the PACI tool	Kaasalainen et al.	2011b
	Physical cues	Abnormal skin colour	Not significant	Response frequency to "What are the signs or indicators of pain in noncommunicative elderly people with dementia?"	Cohen-Mansfield & Creedon	2002
		Swollen joints	Not significant	Response frequency to "What are the signs or indicators of pain in noncommunicative elderly people with dementia?"	Cohen-Mansfield & Creedon	2002
Facial expressions	Brows	Brow lowering	No information	Higher frequencies reported in more painful situations.	Lints-Martindale et al.	2007
			No information	Validity of the marker in the PACI tool	Kaasalainen et al.	2011b
	Cheeks	Cheek raising	No information	Validity of the marker in the PACI tool	Kaasalainen et al.	2011b
			Not significant	Patient was not more likely to have a high pain score when this symptom was present.	Atee et al.	2022
	Eyes	Blinking	No information	Higher frequencies reported in more painful situations.	Lints-Martindale et al.	2007
			Not significant	Marker importance as rated by the nursing staff	Cohen-Mansfield & Creedon	2002
		Closing eyes	No information	Higher frequencies reported in more painful situations.	Lints-Martindale et al.	2007
		Eyes down	No information	Higher frequencies reported in more painful situations.	Lints-Martindale et al.	2007
		Eyes left	No information	Higher frequencies reported in more painful situations.	Lints-Martindale et al.	2007
		Eyes right	No information	Higher frequencies reported in more painful situations.	Lints-Martindale et al.	2007
		Eyes up	No information	Higher frequencies reported in more painful situations.	Lints-Martindale et al.	2007
		Tightening of eyelids	No information	Validity of the marker in the PACI tool	Kaasalainen et al.	2011b

			No information	Higher frequencies reported in more painful situations.	Lints-Martindale et al.	2007
	Jaw	Jaw drop	No information	Higher frequencies reported in more painful situations.	Lints-Martindale et al.	2007
	Lips, mouth	Parting lips	No information	Higher frequencies reported in more painful situations.	Lints-Martindale et al.	2007
		Raising of upper lip	Not significant	correlation between the behaviour item of the OPS-NVI and the presence of orofacial pain according to self-report	van de Rijt et al.	2019
	Whole face	Change in colour	Not significant	Response frequency to “What are the signs or indicators of pain in noncommunicative elderly people with dementia?”	Cohen-Mansfield & Creedon	2002
		Facial expression - specific	No information	Reports of formal caregivers	Kaasalainen et al.	2011a
		Grimacing	No information	Symptom occurrence as associated with pain	Jansen et al.	2017
		Grimacing	No information	Symptom occurrence as associated with pain	Lundin et al.	2021
		Relaxed	Not significant	significant association for the absence of pain	Cervo et al.	2007
		Sudden jerk	No information	Higher frequencies reported in more painful situations.	Lints-Martindale et al.	2007
Mental state / mood	Mood indicators	Depression	No information	Symptom occurrence as associated with pain	Karlsson et al.	2015
Physiology	Breathing	Ease of breathing	Not significant	Inter-rater agreement ("Good indication to judge pain")	van Iersel et al.	2006
	Vital signs - several	Changes in vital signs - general	Not significant	Response frequency to “What are the signs or indicators of pain in noncommunicative elderly people with dementia?”	Cohen-Mansfield & Creedon	2002
			No information	Reports of formal caregivers	Kaasalainen et al.	2011a
			Not significant	Inter-rater agreement ("Good indication to judge pain")	van Iersel et al.	2006
Speech, language and sounds	Inappropriate verbal behavior	Verbal sexual advances	Not significant	No relationship to pain	Husebo, Ballard et al.	2013
	Non-verbal expression	[General]	No information	Reports of formal caregivers	Kaasalainen et al.	2011a
		Making strange noise	Not significant	No relationship to pain	Husebo, Ballard et al.	2013
		Negative vocalizations	No information	Symptom occurrence as associated with pain	Lundin et al.	2021
			No information	Symptom occurrence as associated with pain	Karlsson et al.	2015

	Screaming	Not significant	No relationship to pain	Husebo, Ballard et al.	2013
	Sighing	Not significant	No relationship to pain	Shega et al.	2008
